# Broadband photodetection of intense lasers via exciton-enhanced high-order multiphoton-absorption optoelectronics in 2D hybrid perovskite

**DOI:** 10.1126/sciadv.adt9952

**Published:** 2025-05-23

**Authors:** Yanming Xu, Haojie Xu, Pengfei Zhu, Jinlong Xu, Yantang Huang, Zhihua Sun, Fushan Li, Shining Zhu, Lin Zhou

**Affiliations:** ^1^ College of Physics and Information Engineering, Fuzhou University, Fuzhou 350108, China.; ^2^ National Laboratory of Solid State Microstructures, School of Electronic Science and Engineering, College of Engineering and Applied Sciences, Nanjing University, Nanjing 210093, China.; ^3^ State Key Laboratory of Structure Chemistry, Fujian Institute of Research on the Structure of Matter, Chinese Academy of Sciences, Fuzhou 350002, China.

## Abstract

Broadband photodetection, especially for the high-intensity pulsed lasers, has garnered increasing interest in photophysics and applied sciences driven by the development of pulsed lasers. However, direct broadband photodetection of high-intensity pulsed lasers, with precisely capturing their spatiotemporal properties, has been hampered by low saturation intensity or damage threshold of traditional optoelectronic materials. Here, we demonstrate that strategic enhancement of excitonic effects in two-dimensional (2D) layered hybrid perovskite can enable robust high-order multiphoton absorption (MPA) optoelectronics with achieving strong four-photon absorption (4PA) and five-photon absorption (5PA) nonlinearities as well as efficient electronic properties simultaneously. This effectively overcomes the limitations of mainstream photodetectors. Our approach facilitates direct photodetection and high-precision imaging of high-intensity femtosecond lasers (21.5 GW/cm^2^) across a broad wavelength range of 800 to 2300 nanometer. These results offer valuable insights into advancing high-order nonlinearity-based optoelectronics and provide practical solutions for direct measurement tools of intensive lasers, filling the blank of high-precision characterization of intense-field laser phenomena.

## Teaser

Broadband high-power pulsed-laser photodetection is realized by excitonic high-order multiphoton absorption in layered perovskite.

## INTRODUCTION

Since the invention of Ti:sapphire femtosecond (fs) laser (*1*), high-intensity pulsed lasers have been recognized as revolutionary tools enabling numerous breakthroughs in both fundamental science and advanced technology. Accurate identification of spatiotemporal features is a basic requirement for the applications of high-intensity lasers. However, commercial photodetectors (PDs) typically have low saturation intensities and damage thresholds, which necessitates the use of beam splitting or attenuation elements (e.g., glass plates, diffusers, and filters) in the front of PDs to reduce the laser intensities. Under the excitation of intense field, these optical elements can induce many nonlinear optical effects such as super-continuum spectrum (*2*), harmonic generation (*3*), and nonlinear refraction and self-phase modulation (*4*), thus producing various spatiotemporal distortions ranging from spectral noise (*5*) to frequency chirping (*6*) to self-focusing or diverging (*7*) to beam distortions (*8*), etc. Therefore, developing strategies for direct photodetection of high-intensity pulsed lasers has long been desired.

As a typical nonlinear optical effect in response to intense laser excitation, multiphoton absorption (MPA) enables sub-bandgap optoelectronics by allowing the simultaneous absorption of multiple photons with energies below the bandgap of optoelectronic materials. This unique process facilitates the development of MPA PDs with extremely high saturation intensities. The MPA PDs based on ZnO (*9*), MoS_2_ (*10*), and hybrid perovskites (*11*–*15*) have demonstrated the successful applications of two-photon absorption (2PA) and three-photon absorption (3PA) optoelectronics for detecting intense fs lasers. However, the major limitations of current MPA PDs are their short maximum responsive wavelength (shorter than 1.6 μm) and low maximum responsive intensity (lower than 2.5 GW/cm^2^), which mainly arise from the absence of efficient higher-order MPA optoelectronics. Over the past years, considerable efforts have been made to seek innovative MPA materials to address this issue (*16*–*20*), but it still remains unattainable.

The optoelectronic properties of perovskites have been intensively investigated in the past decade since the first report on perovskite-based PD (*21*). Recently, two-dimensional (2D) layered Ruddlesden-Popper (RP) hybrid perovskites, featuring a natural multilayered quantum well (QW) structure with alternating organic layers (barriers) and inorganic sheets (wells) (*22*), have shown great potential for modulating optical and electronic properties (*23*–*25*). The strong quantum and dielectric confinement effects within these QWs result in large exciton binding energies, allowing for formation of room-temperature stable excitons (*26*). These excitons can serve as a unique type of transition dipoles (*27*–*29*) to enhance optical nonlinearities (*30*). However, realizing the full potential in MPA PDs requires simultaneous optimization of both the MPA nonlinearities of excitons and the electrical properties of carriers. This remains a huge challenge due to the fact that the properties of excitons and carriers are oppositely correlated to the interaction strength of electron-hole pairs (*31*–*33*).

In this work, by carefully designing the QW structure of a 2D RP hybrid perovskite, (BA)_2_(MA)_2_Pb_3_Br_10_ (BMPB, BA = *n*-butylamine and MA = methylamine), we simultaneously actualize strong high-order MPA nonlinearities and efficient electronic properties. The four-photon absorption (4PA) and five-photon absorption (5PA) coefficients of BMPB are 9.1 × 10^−4^ cm^5^ GW^−3^ and 8.7 × 10^−5^ cm^7^ GW^−4^, respectively, almost 3000 times higher than those of mainstream MPA materials (*34*–*38*). This exceptional performance enables construction of a BMPB PD for directly detecting high-intensity fs lasers in a broad wavelength-response range of 800 to 2300 nm. This PD also shows a broad intensity-response range, from 1.2 MW/cm^2^ to 21.5 GW/cm^2^. Furthermore, it exhibits high-precision imaging capabilities for the intrinsic features of high-intensity fs lasers at different spatial modes, exceeding the performance of Si-based charge-coupled device (CCD). These findings highlight the exceptional potential of high-performance MPA PD for accurately characterizing and imaging high-intensity lasers.

## RESULTS

### Mechanism for exciton-enhanced high-order MPA optoelectronics

**Fig. 1.**
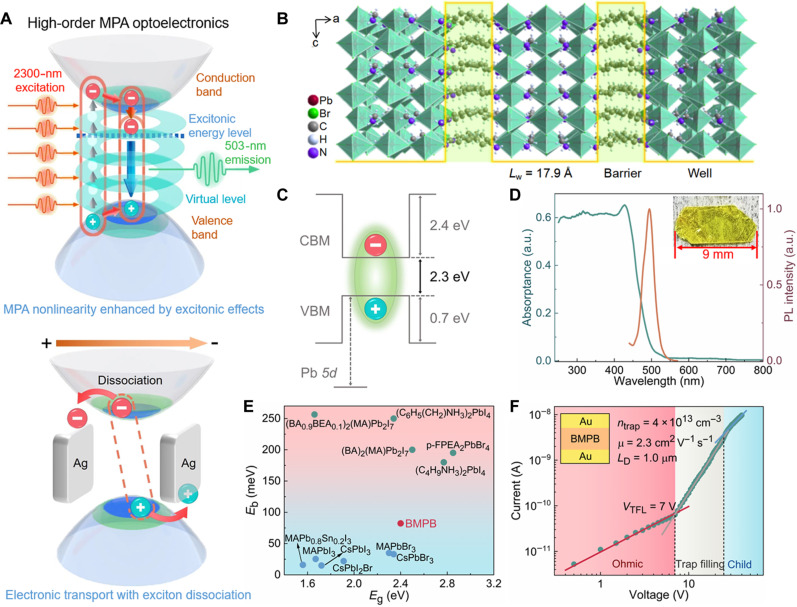
The principle of exciton-enhanced MPA optoelectronics and the physical properties of BMPB crystal. (**A**) Mechanism of excitonic high-order MPA optoelectronics. Top: Schematic illustration of MPA nonlinearity enhanced by excitonic effects; bottom: schematic diagram of the exciton dissociation and charge transport under bias electric field. (**B**) Diagram of the crystal structure for top view in *ac* plane. (**C**) Energy-level scheme evidencing its type-I QW structure. (**D**) Linear optical absorption and steady-state PL emission spectra. The excitation intensity for PL emission is 14 mW/cm^2^ at 405 nm. Inset: Photograph of the as-grown BMPB crystal. (**E**) Comparison of the exciton binding energies ($E_{b}$ = 82.3 meV for BMPB) with typical perovskites. References: MAPbI_3_ (*40*), MAPbBr_3_ (*40*), MAPb_0.8_Sn_0.2_I_3_ (*41*), CsPbI_3_ (*42*), CsPbBr_3_ (*42*), (BA)_2_(MA)Pb_2_I_7_ (*43*), (C_4_H_9_NH_3_)_2_PbI_4_ (*44*), p-FPEA_2_PbBr_4_ (*45*), (C_6_H_5_(CH_2_)_2_NH_3_)_2_PbI_4_ (*25*), and (BA_0.9_PEA_0.1_)_2_(MA)Pb_2_I_7_ (*46*). (**F**) Logarithmic *I-V* curve measured by the SCLC model. Inset: Diagram of the planar-type device structure used for *I-V* measurement. a.u., arbitrary unit.

To fulfill superior MPA optoelectronics, both efficient high-order MPA nonlinearities and electronic transport properties should be realized. In this case, the primary requirement is the generation of high density of excitons to enhance MPA nonlinearities, as the transition dipole moments of excitons can contribute macroscopic nonlinear optical susceptibility (*27*–*30*, *39*). The mechanism for MPA nonlinearities enhanced by exciton dipoles is schematically illustrated in the top of Fig. 1A. Under intense near-infrared (NIR) fs-laser excitation, the valence electrons are excited to the conduction band by simultaneously absorbing multiple NIR photons via virtual intermediate levels, leading to the formation of excitons with pronounced Coulombic interactions. These excitons can act as transition dipoles, providing a large dipole moment (*27*–*29*) to facilitate the MPA process of other valence electrons and generating more exciton dipoles. High-density exciton dipoles will strongly enhance the macroscopic nonlinear optical susceptibilities $\chi^{\left(n\right)}\;\left(n=3,5,7\ldots \right)$ at both lower orders [i.e., $\chi^{\left(3\right)}$ for 2PA] and higher orders [$\chi^{\left(5\right)}$ for 3PA, $\chi^{\left(7\right)}$ for 4PA, and $\;\chi^{\left(9\right)}$ for 5PA], respectively. This necessitates a large exciton binding energy to maintain exciton stability at room temperature. The second requirement is the dissociation of a sufficient number of excitons under the applied electric field to ensure efficient electronic transport properties (bottom of Fig. 1A). To ensure efficient exciton dissociation, the ideal structure of MPA material should be featured by moderate dielectric confinement.

Therefore, achieving a strategic trade-off between MPA nonlinearities and electronic transport is crucial for efficient MPA photodetection. This indicates that the designing of RP hybrid perovskites should obey the following structural principles: (i) A few-layer inorganic sheets for obtaining strong quantum confinement and a large exciton binding energy. (ii) A suitable dielectric contrast between the inorganic and organic layers (i.e., $\varepsilon_{\text{inorg}}/\varepsilon_{\text{org}}$) to achieve moderate dielectric confinement. (iii) Strong interlayer hydrogen bonds for high stability and resistance to damage from high-intensity pulsed lasers.

### Basic characterization of BMPB nonlinear crystal

Based on the above principles, we have designed a RP-type hybrid perovskite, BMPB, in which the inorganic sheets of corner-sharing PbBr_6_ octahedra link to organic BA bilayers via N─H…Br hydrogen bonds along the *a* axis, while MA^+^ cations reside in the perovskite cavities (Fig. 1B). Density functional theory (DFT) calculations reveal that BMPB has a direct bandgap of $E_{g}$ = 2.3 eV (fig. S1A). Partial density of states analyses indicate that its valence band maximum and conduction band minimum originate from the Br-4p and Pb-6p states, respectively, confirming the role of the inorganic framework in determining its bandgap (fig. S1B). This evidences its type-I quantum structure, with inorganic sheets serving as the wells and organic layers acting as the barriers. The 1.8-nm-thick well layer enables strong quantum confinement (Fig. 1C).

Large BMPB crystals (inset in Fig. 1D) grown from an aqueous solution (see more details in Materials and Methods) show a series of diffraction peaks corresponding to the (200) family of crystal planes in the x-ray diffraction (XRD) pattern (fig. S1C), validating the highly oriented alignment perpendicular to its crystallographic *a* axis and thus high-quality nature of the crystals. The crystals exhibit an absorption cutoff at 520 nm and a sharp photoluminescence (PL) peak around 500 nm (Fig. 1D), closely matching the calculated bandgap. Temperature-dependent PL spectra reveal an exciton binding energy of $E_{b}$ = 82.3 meV (note S2), which is higher than those of most 3D perovskites (*40*–*42*) but lower than those of some mainstream 2D layered perovskites (*25*, *43*–*46*) (Fig. 1E and table S1). This difference is primarily ascribed to the moderate dielectric contrast between inorganic and organic parts ($\varepsilon_{\text{inorg}}/\varepsilon_{\text{org}}$ = 6.0 with $\varepsilon_{\text{inorg}}$= 32.3 and $\varepsilon_{\text{org}}$ = 5.4), larger than those in 3D perovskites but smaller than most 2D perovskite counterparts (*47*, *48*). We anticipate that this strategic modification of excitonic effects will not only stabilize exciton dipoles at room temperature to enhance MPA nonlinearities but also allow the dissociation of abundant excitons under an electric field to enable good charge transport properties.

Ultraviolet photoelectron spectroscopy (*49*, *50*) determines BMPB to be p-type semiconductor (fig. S2). As shown in Fig. 1F, the in-plane carrier diffusion properties of BMPB are determined with a planar-type device structure based on the space charge–limited current (SCLC) method (*51*). It exhibits three distinct regions in the logarithmic current-voltage (*I*-*V*) curve: linear Ohmic region, trap-filling region, and quadratic Child’s region. The trap-state density is estimated to be $n_{\text{trap}}$ = 4 × 10^13^ cm^−3^ from the trap-filling region, which is three orders of magnitude lower than that of 2D perovskite (BA)_2_(MA)_3_Pb_4_I_13_ (~10^16^ cm^−3^) (*52*) and comparable with that of 3D perovskite MAPbBr_3_ (1.1 × 10^13^ cm^−3^) (*53*). In the trap-free Child’s region, the carrier mobility is derived to be μ = 2.3 cm^2^ V^−1^ s^−1^, which surpasses the hybrid perovskite films of MAPbI_3_ (1.3 cm^2^ V^−1^ s^−1^) (*54*) and MAPbBr_3_ (0.26 cm^2^ V^−1^ s^−1^) (*55*). Combined this with the PL lifetime (τ = 217.2 ns) obtained from the time-resolved PL spectra (fig. S1D), the in-plane diffusion length ($L_{D}$) is calculated to be ~1.0 μm, which is comparable to those of MAPbI_3_ (1.8 μm) (*56*), MAPbBr_3_ (1.3 μm) (*56*), and FAPbI_3_ crystals (1.7 μm) (*57*), confirming the efficient charge transport properties of BMPB.

### Broadband MPA nonlinearities of BMPB crystal

**Fig. 2.**
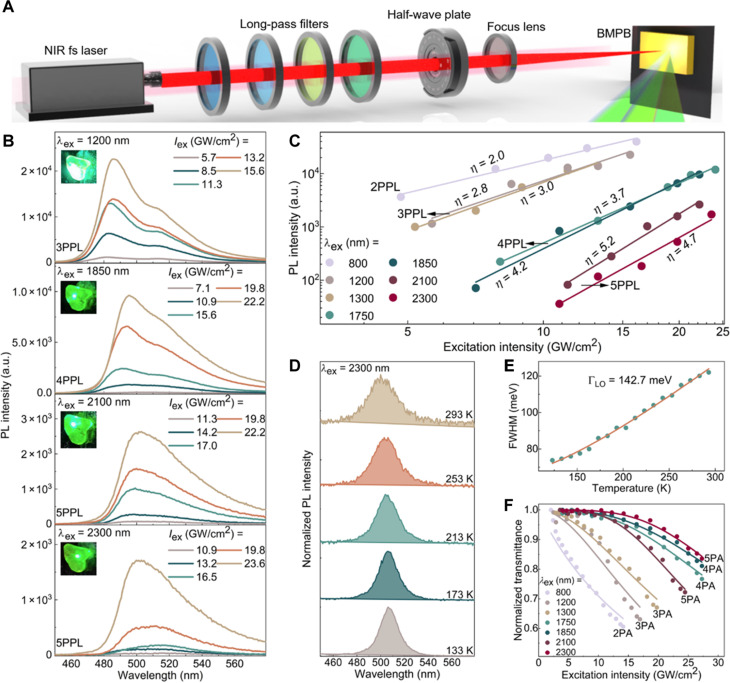
MPA nonlinear optical properties of BMPB crystal. (**A**) Schematic diagram of the experimental setup for MPA-excited PL measurement. (**B**) 3PPL ($\lambda_{\text{ex}}$ = 1200 nm), 4PPL ($\lambda_{\text{ex}}$ = 1850 nm), and 5PPL ($\lambda_{\text{ex}}$ = 2100 and 2300 nm) spectra at different excitation intensities. Insets: photographs of *n*PPL emission from BMPB crystal. (**C**) Logarithmic plot of the *n*PPL intensity dependence on excitation intensity at different wavelengths. Solid lines: fitting with the power-law relation $I_{n\text{PPL}}\propto I_{\text{ex}}^{\eta }$. (**D**) Normalized 5PPL spectra measured at five temperatures. (**E**) FWHM of 5PPL spectra as a function of temperature. Solid line: fitting curves for $\Gamma_{\text{LO}}$ extraction. (**F**) Normalized transmittance as a function of excitation intensity for 2PA, 3PA, 4PA, and 5PA processes. Solid lines: fitting curves for MPA absorption coefficients.

The MPA nonlinearities of BMPB crystal under broadband fs-laser excitation were studied by measuring the MPA-excited upconversion process. Because of the enhanced excitonic nonlinearities in BMPB, strong *n*PA-excited PL (*n*PPL, *n* = 2, 3, 4, and 5) was observed across a broad NIR range of excitation wavelengths ($\lambda_{\text{ex}}$) from 800 to 2300 nm with the experimental setup presented in Fig. 2A. The recorded PL spectra depending on excitation intensity ($I_{\text{ex}}$) are depicted in Fig. 2B ($\lambda_{\text{ex}}$ = 1200, 1850, 2100, and 2300 nm) and fig. S5 ($\lambda_{\text{ex}}$ = 1300 and 1750 nm). A gradual red-shift of PL peaks from 486 to 503 nm can be seen with increasing $\lambda_{\text{ex}}$ due to the reabsorption effect (*58*, *59*). This effect also generates an additional low-energy peak (~520 nm), a phenomenon commonly observed in the MPA-excited PL emission of perovskites (*60*–*62*). Furthermore, this peak diminishes as the excitation wavelength increases from 1200 to 2300 nm (Fig. 2B), corresponding to an increase of MPA order from 3PA to 5PA. This behavior can be attributed to the reduction in MPA-induced PL strength, as higher-order MPA nonlinearities significantly lower the MPA excitation efficiency, thereby weakening the reabsorption effect.

From the nonlinear curves of *n*PPL intensity ($I_{n\text{PPL}}$) as a function of $I_{\text{ex}}\;$ (Fig. 2C), the fitting slopes in the logarithmic plot meet η≈n dependence for *n*PA process, in accordance with the power law for perturbative nonlinear process, i.e., $I_{n\text{PPL}}\propto I_{\text{ex}}^{n}$ (see Materials and Methods for theoretical derivation). These results confirm the orders of MPA nonlinearities in BMPB: 2PA at $\lambda_{\text{ex}}$ = 800 nm, 3PA at $\lambda_{\text{ex}}$ = 1200 and 1300 nm, 4PA at $\lambda_{\text{ex}}$ = 1750 and 1850 nm, and 5PA at $\lambda_{\text{ex}}$ = 2100 and 2300 nm, respectively.

Further investigation into the temperature-dependent PL spectroscopy was conducted to determine the exciton-phonon coupling strength (*63*). As shown in Fig. 2D, the 5PPL spectra ($\lambda_{\text{ex}}$ = 2300 nm) exhibit continuous broadening with increasing the temperature from 133 to 293 K. The exciton-longitudinal optical-phonon coupling strength ($\Gamma_{\text{LO}}$) is derived to be 142.7 meV by fitting the full width at half maximum (FWHM) of variable-temperature 5PPL spectra (Fig. 2E and see note S4 for details). This value is larger than those of typical 3D perovskites such as FAPbI_3_ (34.8 meV) (*64*) and FAPbBr_3_ (45.4 meV) (*65*) but smaller than some similar 2D perovskites such as (BA)_2_(MA)Pb_2_I_7_ (275 meV) (*66*) and (PEA)_2_PbCl_4_ (261.5 meV) (*67*). This result indicates a moderate strength of exciton localization within the QWs, which will facilitate the electronic transport of photo-generated carriers.

To quantitatively characterize the MPA performance of BMPB, the open aperture (OA) Z-scan measurement was performed (note S5). The intensity dependence of normalized transmittance is illustrated in Fig. 2F ($\lambda_{\text{ex}}$ = 800 to 2300 nm). The extracted absorption coefficients for each MPA process are listed in Table 1 (see Materials and Methods for details). It can be found that the MPA absorption coefficients of BMPB are notably higher than those of typical inorganic semiconductors and organic chromophores and are comparable with typical all-inorganic or hybrid perovskites (table S2).

**Table 1.** MPA absorption coefficients of BMPB crystal. β, γ, δ, and φ: 2PA, 3PA, 4PA, and 5PA absorption coefficient, respectively.

Excitation wavelength (nm)	MPA order	Absorption coefficient
800	2	β = 2.1 cm GW^−1^
1200	3	γ = 0.11 cm^3^ GW^−2^
1300	3	γ = 0.07 cm^3^ GW^−2^
1750	4	δ = 9.1 × 10^−4^ cm^5^ GW^−3^
1850	4	δ = 6.5 × 10^−4^ cm^5^ GW^−3^
2100	5	φ = 8.7 × 10^−5^ cm^7^ GW^−4^
2300	5	φ = 2.2 × 10^−5^ cm^7^ GW^−4^

### MPA-excited sub-bandgap photodetection on broadband fs lasers

**Fig. 3.**
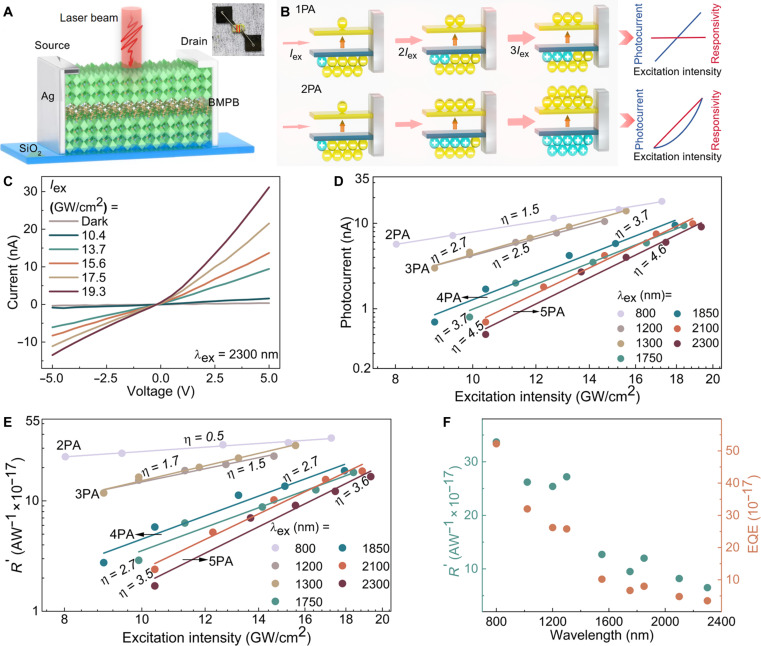
MPA-based sub-bandgap photodetection of broadband NIR fs laser based on BMPB crystal. (**A**) Schematic illustration of the MPA PD based on BMPB. Inset: photograph of the PD; red solid lines: channel area. (**B**) Comparison of the photo-carrier generation mechanism between 1PA and 2PA, as well as the photocurrent and responsivity dependence on excitation intensity. (**C**) *I*-*V* curves measured at different excitation intensities for 5PA ($\lambda_{\mathrm{ex}}$ = 2300 nm) photodetection. (**D**) Logarithmic plot of MPA photocurrent dependence on excitation intensity ($V_{b}$ = 2 V). Solid lines: fitting with the power-law relation $I_{\mathrm{ph}}\propto I_{\mathrm{ex}}^{\eta }$, where the extracted η meets η≈n for *n*PA (*n* = 2, 3, 4, and 5). (**E**) Logarithmic plot of the peak-power responsivity dependence on excitation intensity. Solid lines: fitting with the power-law relation $R\prime \propto I_{\mathrm{ex}}^{\eta }$, where the extracted η meets η≈n−1 for *n*PA. (**F**) Peak-power responsivity and EQE dependence on excitation wavelength ($V_{b}$ = 2 V and $I_{\mathrm{ex}}$ = 14.2 GW/cm^2^).

The simultaneous realization of strong MPA nonlinearities and efficient electronic transport in BMPB crystal provides opportunities for the direct detection of broadband high-intensity fs lasers. Figure 3A illustrates the as-fabricated PD by transferring 0.8-mm-thick BMPB crystal on a SiO_2_ substrate, with a channel length of 1 mm and a width of 5 mm. A fs-laser beam with a diameter of ~0.6 mm was irradiated on the channel. Notably, as depicted in Fig. 3B, the behavior of BMPB PD based on sub-bandgap MPA is quite distinct from that of conventional PDs based on above-bandgap one-photon absorption (1PA). In conventional PDs, the photocurrent ($I_{\text{ph}}$) shows a linear dependence on $I_{\text{ex}}$, and the responsivity (R) remains nearly constant (*68*, *69*) (top of Fig. 3B). In contrast, for MPA PDs, the number of photo-generated carriers increases by the *n*-th power with $I_{\text{ex}}$, leading to nonlinear relationships of $I_{\text{ph}}\propto I_{\text{ex}}^{n}\;$and $R\propto I_{\text{ex}}^{n-1}$. For example, in the case of 2PA photodetection (bottom of Fig. 3B), the number of photo-generated carriers is proportional to $I_{\text{ex}}^{2}$, resulting in a quadratic growth of $I_{\text{ph}}$ and a linear growth of R with $I_{\text{ex}}$.

The experimentally measured *I*-*V* curves under the excitation of 405-nm continuous-wave laser (for 1PA process) and fs lasers in the range of 800 to 2300 nm (for 2PA-5PA processes) are shown in Fig. 3C (for $\lambda_{\text{ex}}$ = 2300 nm) and fig. S9 (for $\lambda_{\text{ex}}$ = 405, 800, 1300, 1750, 1850, and 2100 nm), respectively. The photocurrent shows a monotonous increase trend with $I_{\text{ex}}$ due to the combined effects of strong MPA and efficient carrier transport. Furthermore, in the logarithmic plot of $I_{\text{ph}}∼I_{\text{ex}}$ under a bias voltage of $V_{b}$ = 2 V (fig. S10 for $\lambda_{\text{ex}}$ = 405 nm; Fig. 3D for $\lambda_{\text{ex}}$ = 800 to 2300 nm), the fitting slope for *n*PA-excited photocurrent is slightly lower than the *n* value. This suggests that a portion of carriers recombines during the transport process, while the generation of photocurrent is primarily dominated by MPA effects, consistent with observations reported in other MPA-based photodetection studies (*11*, *12*, *14*).

To evaluate the MPA-based photodetection behavior, we described the responsivity (R′) related to fs-pulse peak power ($P_{\text{peak}}$) as $R\prime =I_{\text{ph}}/P_{\text{peak}}$. In this sense, the external quantum efficiency (EQE) can be described as $\text{EQE}=R\prime \mathrm{hc}/q\lambda_{\text{ex}}$, where h is Planck’s constant, c is the light speed in vacuum, and q is the elementary charge. Based on the experimental data in Fig. 3D, the obtained R′ and EQE for each-order MPA process are depicted in Fig. 3E and fig. S11, respectively. The dependence of R′ and EQE on excitation intensity is well described by the power-law relation, i.e., $R\prime \propto I_{\text{ex}}^{n-1}$ and $\text{EQE}\propto I_{\text{ex}}^{n-1}$, in agreement with the aforementioned MPA mechanism. For instance, with $I_{\text{ex}}$ of 2300-nm fs pulses varying from 10.4 to 19.3 GW/cm^2^, R′ increases from 1.7 × 10^−17^ to 1.7 × 10^−16^ AW^−1^, and EQE increases from 9.0 × 10^−18^ to 9.0 × 10^−17^. In addition, with $\lambda_{\text{ex}}$ increasing from 800 to 2300 nm (under $V_{b}$ = 2 V and $I_{\text{ex}}$ = 14.2 GW/cm^2^), R′ decreases from 3.4 × 10^−16^ to 6.5 × 10^−17^ AW^−1^, and EQE decreases from 5.2 × 10^−16^ to 3.5 × 10^−17^ (Fig. 3F). These behaviors mainly arise from the fact that MPA nonlinearities are weaken with increasing MPA orders and excitation wavelengths, in accordance with the variation of absorption coefficients presented in Table 1. Note that the MPA PD performance does not exhibit obvious dependence on BMPB crystal thickness above 0.1 mm nor on light polarization (note S7).

**Fig. 4.**
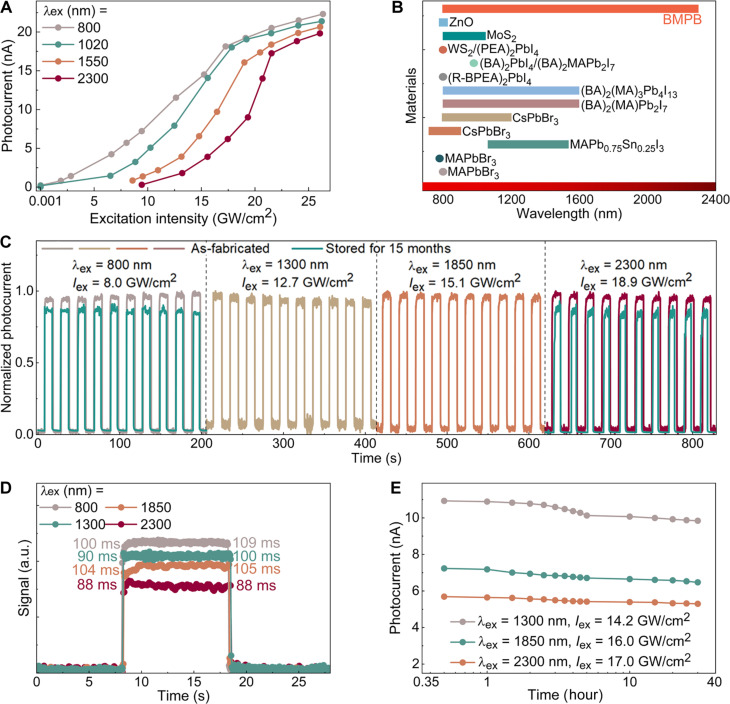
Comparison of intensity/wavelength-response range and temporal/stability measurement for BMPB PD. (**A**) Photocurrent dependence on excitation intensity at different excitation wavelengths ($V_{b}$ = 2 V). (**B**) Wavelength-response range of BMPB compared with some other MPA-based PDs. References: MAPbBr_3_ (*11*), MAPbBr_3_ (*72*), MAPb_0.75_Sn_0.25_I_3_ (*73*), CsPbBr_3_ (*12*), CsPbBr_3_ (*74*), (BA)_2_(MA)Pb_2_I_7_ (*13*), (BA)_2_(MA)_3_Pb_4_I_13_ (*13*), (R-BPEA)_2_PbI_4_ (*14*), (BA)_2_PbI_4_/(BA)_2_MAPb_2_I_7_ (*15*), WS_2_/(PEA)_2_PbI_4_ (*75*), MoS_2_ (*10*), and ZnO (*9*). (**C**) Normalized photocurrent on-off switching cycles ($V_{b}$ = 2 V) of the as-fabricated BMPB PD ($\lambda_{\mathrm{ex}}$ = 800, 1300, 1850, and 2300 nm) and stored in a vacuum box for 15 months ($\lambda_{\text{ex}}$ = 800 and 2300 nm). (**D**) Rise and fall times ($\lambda_{\text{ex}}$ = 800, 1300, 1850, and 2300 nm) of BMPB PD. (**E**) Long-term stability of photocurrent at $\lambda_{\text{ex}}$ = 1300, 1850, and 2300 nm for 30 hours ($V_{b}$ = 2 V).

For broadband high-intensity fs*-*laser photodetection, the intensity- and wavelength-response ranges are the crucial metrics of a MPA PD. Figure 4A depicts the intensity-response spanning of the BMPB PD across the broadband NIR range of 800 to 2300 nm, showing a broad intensity-response range with low thresholds (1.2 MW/cm^2^ at $\lambda_{\text{ex}}$ = 800 nm and 3.2 MW/cm^2^ at $\lambda_{\text{ex}}$ = 1020 nm) and high responsive intensity of ~21.5 GW/cm^2^ across all wavelengths. Furthermore, the intensity-response ranges for different wavelengths are extracted and compared with other representative MPA PDs (fig. S12). The intensity-response threshold at 800 to 1020 nm is significantly lower than the fs-laser–induced retinal damage threshold of ~10 MW/cm^2^ (*70*, *71*), which endows BMPB with great potential for applications in fs-laser safety protection. Note that the threshold measurement at the wavelengths beyond 1020 nm is limited by our fs-laser system: The 800- and 1020-nm pulses are generated by both 80-MHz Ti:sapphire mode-locked oscillator and 5-kHz optical parametric chirped-pulse amplifier (OPCPA), but the 1550- and 2300-nm pulses can only be generated by the high–pulse-energy OPCPA (see more details in Materials and Methods). Therefore, the thresholds for 1550 and 2300 nm maybe much lower than those we measured. Even though, this represents the broadest intensity- and wavelength-response ranges for MPA PDs to the best of our knowledge (*9*–*15*, *72*–*75*) (Fig. 4B, fig. S12B, and see the detailed data in tables S3 and S4). The damage threshold as a function of wavelength was measured as shown in fig. S13. The damage threshold slightly decreases from 33.9 to 32.9 GW/cm^2^ with increasing the excitation wavelength from 800 to 2300 nm, which may be attributed to the reduction of photon energy.

The temporal photocurrent response and long-time stability are another important metrics for PDs. Under periodic on-off switching of the fs*-*laser irradiation, the BMPB PD exhibits stable and reproducible photo-switching behavior across the broad NIR range as shown in Fig. 4C ($\lambda_{\text{ex}}$ = 800, 1300, 1850, and 2300 nm) and fig. S14 ($\lambda_{\text{ex}}$ = 1200, 1750, and 2100 nm). Both the rise and fall times at all the wavelengths are ~100 ms with minimal fluctuation (Fig. 4D). The large exciton binding energy of BMPB prolongs the exciton lifetime and thus leads to a relatively long response time. The response time is expected to improve by reducing the channel length or by constructing a heterostructure with other band-matched materials.

Figure 4E demonstrates the long-time stability of the BMPB PD over 30 hours under intense fs*-*laser irradiation with intensity exceeding 14 GW/cm^2^, without any encapsulation. The absence of noticeable photocurrent degradation suggests its good stability for practical application. In addition, after being stored in a vacuum box for 15 months, this PD still exhibits stable on-off photo-switching behavior. The photocurrent maintains ~91 and ~93% of the initial states at $\lambda_{\text{ex}}$ = 800 and 2300 nm, respectively (Fig. 4C), benefiting from the high-stability structural design of BMPB. Enhanced device stability for practical application will be realizable by encapsulating the PD by polymer film or hexagonal boron nitride (*76*, *77*). Given that perovskite is emerging as a key building block for the next-generation optoelectronics, these excellent performances may boost its potential for high-performance device applications by virtue of the strong high-order MPA optoelectronics.

### Potential applications for accurate imaging of broadband fs lasers

**Fig. 5.**
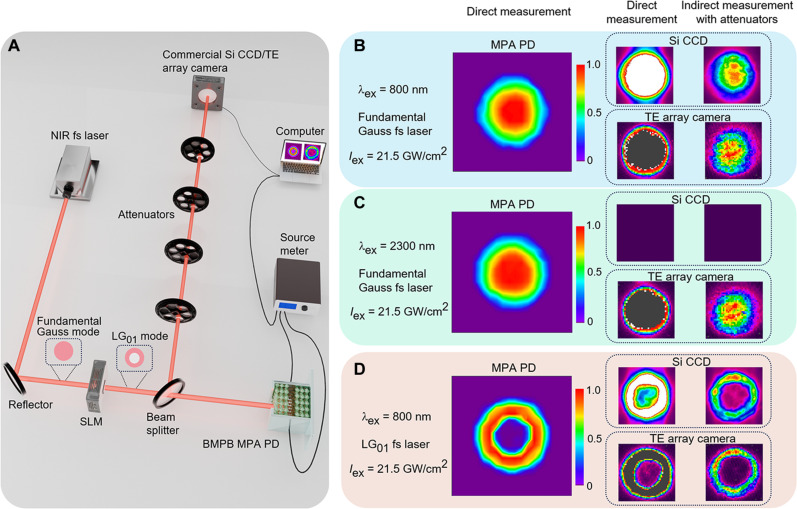
Spatial imaging on the broadband NIR fs-laser beams. (**A**) Schematic illustration of the imaging system based on either the MPA PD or the commercial imaging device. (**B** and **C**) Left: Direct measurement of the fs*-*laser beam with fundamental Gauss mode through the MPA PD at $\lambda_{\text{ex}}$ = 800 and 2300 nm, respectively. Middle: Direct measurement through the commercial Si CCD or TE array camera at $\lambda_{\text{ex}}$ = 800 and 2300 nm, respectively. Right: Indirect measurement through commercial imaging devices with additional attenuation at $\lambda_{\text{ex}}$ = 800 and 2300 nm, respectively. (**D**) Direct and indirect measurements of the fs*-*laser beam with LG_01_ mode at $\lambda_{\text{ex}}$ = 800 nm.

The exceptional photodetection performance of the BMPB PD encourages us to further investigate its practical application in spatial imaging of the broadband high-intensity fs lasers. As illustrated in Fig. 5A, the PD is mounted on a 2D mobile platform, allowing continuous movement in the plane perpendicular to the laser beam (i.e., *xy* plane). The light sources are fs lasers with fundamental Gauss modes at 800 and 2300 nm to verify the broadband imaging capability of the MPA PD. To verify the imaging accuracy of the BMPB PD, the 800-nm fs laser was further modulated into a ring-shaped vortex beam [first-order Laguerre-Gauss (LG_01_) mode] using a spatial light modulator (SLM). The generated photocurrent responding to each spatial position of the laser beam at *xy* plane was recorded by a source meter, followed by conversion of the position-resolved photocurrent into a 2D intensity-distribution imaging with 12 × 12 pixels. Figure 5 (B to D) displays the obtained images for the three fs-laser beams, indicating reliable characterization on the intensity distribution of intense fs lasers at different wavelengths and spatial modes, even at intensities up to 21.5 GW/cm^2^.

For comparison, we used commercial Si-based CCD and LiTaO_3_-based TE array camera to image the same fs-laser beams with results showed in Fig. 5 (B to D). The Si-based CCD, limited by its low saturation threshold and narrow spectral response, experiences strong response saturation when directly exposed to the high-intensity 800-nm beam and has no response to 2300-nm beam. Although the TE array camera has the good capability in broadband response, it also has strong response saturation, hindering its applications in directly imaging intense fs lasers. To address these issues, a series of optical attenuators are usually placed in the front of these devices. However, as aforementioned, the high-intensity field on the attenuators can excite various nonlinear optical effects and thus introduce severe beam distortions, leading to substantial errors in imaging the intrinsic laser features. The comparison to InGaAs photodiode also verifies the exceptional performance of MPA PD for intense fs-laser imaging at 900 and 1700 nm (note S8). Therefore, the BMPB PD presents an intriguing solution to this long-standing limitation on accurately detecting and imaging strong-field phenomena. Furthermore, enhanced accuracy, resolution, and stability are expected by further fabricating CCD array based on BMPB.

## DISCUSSION

In summary, we have demonstrated a promising pathway for high-precision detection on broadband high-intensity lasers by simultaneously realizing strong high-order MPA nonlinearities and efficient electronic transport properties in the 2D layered hybrid perovskite BMPB. Based on the robust 2PA-5PA optoelectronic performance, the fabricated BMPB PD exhibits a broad high-intensity-response range and high saturation intensities across a broad NIR spectral region (800 to 2300 nm), which surpasses current state-of-the-art PDs. This PD is further applied to accurately image the features of broadband high-intensity fs lasers, highlighting the promising potential of BMPB in CCD-array fabrication with high accuracy and resolution. These findings not only provide a feasible strategy for accurately investigating high-intensity laser field but also expand the potential applications of 2D hybrid perovskites in advanced optoelectronic technologies.

## MATERIALS AND METHODS

### Synthesis and growth of BMPB bulk crystals

During chemical synthesis of BMPB raw material, *n*-butylamine (99%), methylamine (40% in H_2_O), and lead acetate trihydrate [Pb(Ac)_2_, 99.5%] were stoichiometrically dissolved in the concentrated solution of hydrobromic acid (HBr, 48%). Large-sized yellow crystals were obtained after 1 week by the temperature cooling method.

### Basic characterization of BMPB crystal

The single crystal x-ray diffraction was performed on Bruker D8 diffractometer with Mo *K*α radiation (λ = 0.77 Å). Crystal structure of BMPB crystal was solved by the direct method and confirmed by the full-matrix least-squares refinements on *F*^2^ using the SHELXTL software packing. All the non–H atoms were refined anisotropically, and H atoms were generated by geometrical method and refined using the Olex2 software. The linear absorption spectra were measured at room temperature on the PerkinElmer Lambda 900 ultraviolet-visible-NIR spectrometer. The PL measurements were performed on the Edinburgh FLS920 fluorescence spectrometer. For the dielectric constant measurement, silver paste was deposited on the surfaces of the bulk crystals, and the impedance analyses were performed using the TongHui TH2828A impedance analyzer.

### Fabrication of MPA-based PD

The planar-type PD was fabricated on the SiO_2_ substrate, and surface of bulk crystals was cleaned by nitrogen flow beforehand. Silver paste was deposited on the two terminals of crystal surface to form a pair of Ag electrodes. The channel length and width are 1 and 5 mm, respectively. After the preparation of electrodes, the device was annealed at 70°C in the vacuum atmosphere for 2 hours.

### Steady-state PL measurements

During the 1PPL measurement, a continuous-wave laser ($\lambda_{\text{ex}}$ = 405 nm) was used as the incident excitation light. For the MPA-related optical measurements, both Ti:sapphire mode-locked oscillator (Chameleon, Coherent; ~120 fs, 80 MHz, 680 to 1020 nm) and OPCPA (Topas-C, Coherent; 5 kHz, 800 to 2300 nm) were used as the laser sources. In all the experiments, the crystal samples were rotated repeatedly to ensure the consistency between their *c* axes and the polarization direction. The output laser of OPCPA was filtered by suitable long-pass filters to remove the light at undesired wavelengths. Two continuously variable neutral density filters were used to adjust the intensity of excitation laser. For the steady-state PL measurements, a circular lens with the focal length of 80 mm was used to focus the laser vertically onto the bulk crystals with the thickness of ~0.5 mm. The PL signals were collected by an optical fiber and coupled to the spectrometer (QE Pro, Ocean Optics) in a reflection spectroscopy geometry. The fiber port was fixed as close as to the sample with a small angle of ~10°. For the temperature-dependent PL measurements, the samples were put in a heating stage, and the temperature was set from 123 to 293 K with the heating rate of 3.4 K/min.

### OA Z-scan measurements

The incident laser beam was divided into two parts by a beam splitter. One part was directed into a power detector (*D*_R_) as the reference, while the other part was focused onto the bulk crystals (the thickness ~0.2 mm) by a circular lens with the focal length of 200 mm. The beam transmitted through the samples was detected by another power detector (*D*_S_) as the signal. The incident beam was propagating along the crystallographic *a* axis, and the samples moved along the propagating direction of the beam, i.e., *z* axis. The transmittance was recorded as a function of the sample position (z). As the incident power can be regarded as a constant, the sample will be subjected to various peak intensity I(z) at different position and subsequently the change of transmittance. The diameters (d) of the laser beams at different position were measured through knife-edge scans as shown in fig. S7.

### Photodetection measurements

The *I*-*V* and *I*-*t* measurements on the MPA-based PD were performed using a Keithley 2450 source-meter controlled by the software developed by Wuhan Zeal Young Technology Co. Ltd. A circular lens with the focal length of 200 mm was used to focus the laser vertically onto the device channel. The diameter of the focused laser beam is ~0.6 mm.

### Imaging on the broadband fs-laser beams

To characterize the spatial distribution of light intensity for the fs-laser beams with fundamental Gauss mode, the MPA-based PD was fixed on a 2D mobile platform and moved continuously in the plane perpendicular to the fs-laser beam (i.e., *xy* plane). The photocurrent at each point was recorded by a Keithley 2450 source meter, and then the position-resolved photocurrent can be converted into a 2D contrast mapping. For comparison, a commercial InGaAs photodiode (DET10D/M, Thorlabs) was used to measure the position-resolved photo response, and the voltage at each point was recorded by a high-speed oscilloscope (MDO3014, Tektronix). In addition, commercial Si-based CCD (LBP2-IR2, Newport) and LiTaO_3_-based TE array camera (PY-III-C-B, Spiricon) were also used to record the intensity distribution for the fs*-*laser beams. For the measurement of the fs beam with LG_01_ mode, a SLM (F4320, CAS Microstar) was used to convert the fundamental Gauss mode into LG_01_ mode.

### Band structure calculations

First-principles DFT calculations were performed using the plane-wave pseudopotential method. The exchange-correlation potential was calculated using Perdew-Burke-Ernzerhof for solids functional within the generalized gradient approximation. The core-electron interactions were described by the norm-conserving pseudopotential.

### Derivation of the MPA strength dependence on excitation intensity

As a nonlinear optical effect, the MPA process can be described by the following expression

$$\frac{\mathrm{dI}\left(z\right)}{\mathrm{dz}}=-\alpha_{1}I\left(z\right)-\alpha_{2}I^{2}\left(z\right)-\alpha_{3}I^{3}\left(z\right)-\alpha_{4}I^{4}\left(z\right)-\alpha_{5}I^{5}\left(z\right)-\cdots$$ (1)

where I(z) is the local intensity of incident laser beam propagating along the *z*-axis, and z is the propagation distance of light in the medium. The parameters $\alpha_{1}$, $\alpha_{2}$, $\alpha_{3}$, $\alpha_{4}$, and $\alpha_{5}$ are one-, two-, three-, four-, and five-photon absorption coefficients, respectively. At a certain photon frequency ν, only the *n*PA process satisfying Eq. 1 is available, and then the following relation can be obtained as

$$\frac{\mathrm{dI}\left(z\right)}{\mathrm{dz}}=-\alpha_{n}I^{n}\left(z\right)$$ (2)

The transmitted laser intensity can be obtained by integration of Eq. 2

$$I\left(L_{0}\right)=\frac{I_{0}}{{\left[1+\left(n-1\right)\alpha_{n}L_{0}I_{0}^{n-1}\right]}^{1/\left(n-1\right)}}$$ (3)

where $I_{0}$ is the laser intensity at the entrance of the sample and $L_{0}$ is the sample thickness. The laser intensity change caused by MPA is as follows

$$\Delta I=I_{0}-I\left(L_{0}\right)=I_{0}\left\{1-\frac{1}{{\left[1+\left(n-1\right)\alpha_{n}L_{0}I_{0}^{n-1}\right]}^{1/\left(n-1\right)}}\right\}$$ (4)

When $\left(n-1\right)\alpha_{n}L_{0}I_{0}^{n-1}\ll 1$, an approximation can be made

$$∆I\approx \left(n-1\right)\alpha_{n}L_{0}I_{0}^{n}$$ (5)

Since the population of excited-state electrons is proportional to the laser intensity, so if we assume that the MPA-induced PL intensity is proportional to the population of excited-state electrons, then the following relation can be obtained as

$$I_{\text{PL}}\propto ∆I\approx \left(n-1\right)\alpha_{n}L_{0}I_{0}^{n}$$ (6)

This indicates that for *n*PA process, the up-conversion PL intensity grows as *n*th power with the excitation intensity. At the same time, if we assume that the photocurrent is proportional to the population of excited-state electrons, a similar relation can be obtained as

$$I_{\text{ph}}\propto ∆I\approx \left(n-1\right)\alpha_{n}L_{0}I_{0}^{n}\;$$ (7)

This suggests that for *n*PA photodetection, the photocurrent grows as *n*th power with the excitation intensity. Therefore, the responsivity can be expressed as follows

$$R=\frac{I_{p}}{P}\propto \left(n-1\right)\alpha_{n}L_{0}I_{0}^{n-1}$$ (8)

where P is the incident laser power. This suggests that for *n*PA photodetection, the responsivity grows as (*n* − 1)-th power with the excitation intensity.

For the above-bandgap 1PA photodetection, the linear absorption process can be described as follows

$$\frac{\mathrm{dI}\left(z\right)}{\mathrm{dz}}=-\alpha_{1}I\left(z\right)$$ (9)

The transmitted laser intensity is as follows

$$I\left(L_{0}\right)=I_{0}e^{-\alpha_{1}L_{0}}$$ (10)

So that the following relation can be obtained as

$$I_{\text{ph}}\propto \Delta I=I_{0}-I\left(L_{0}\right)=I_{0}\left(1-e^{-\alpha_{1}L_{0}}\right)$$ (11)

This suggests that for 1PA photodetection, the photocurrent is proportional to the excitation intensity. Further, the responsivity can be obtained as

$$R=\frac{I_{p}}{P}\propto 1-e^{-\alpha L_{0}}$$ (12)

This means that for 1PA photodetection, the responsivity is a constant value invariable with the excitation intensity.

### Determination of the MPA absorption coefficients

Based on Eq. 3, the transmittance in *n*PA process can be expressed as follows

$$T=\frac{I\left(L_{0}\right)}{I_{0}}=\frac{1}{{\left[1+\left(n-1\right)\alpha_{n}L_{0}I_{0}^{n-1}\right]}^{1/\left(n-1\right)}}$$ (13)

Note that the value of $I_{0}$ varies with the position z along the *z*-axis direction, so that the transmittance at the different positions can be measured experimentally. By fitting the experimental data with Eq. 13, the *n*PA absorption coefficients can be determined.

## Supplementary Materials

### This PDF file includes:

Notes S1 to S8 Figs. S1 to S17 Tables S1 to S4 References
