# Supplementary material for: Broadband photodetection of intense lasers via exciton-enhanced high-order multiphoton-absorption optoelectronics in 2D hybrid perovskite
Source: Sci Adv. 2025 May 23;11(21):eadt9952. doi: 10.1126/sciadv.adt9952 (PMC12101496; doi:10.1126/sciadv.adt9952)
Supplement: Supplementary file 1 — Notes S1 to S8 Figs. S1 to S17 Tables S1 to S4 References [file sciadv.adt9952_sm.pdf]

Science Advances
AAAS

Supplementary Materials for

Broadband photodetection of intense lasers via exciton-enhanced high-order multiphoton-absorption optoelectronics in 2D hybrid perovskite

Yanming Xu *et al.*

Corresponding author: Jinlong Xu, longno.2@163.com; Zhihua Sun, sunzhihua@fjirsm.ac.cn; Fushan Li, fsli@fzu.edu.cn; Lin Zhou, linzhou@nju.edu.cn

Sci. Adv. **11**, eadt9952 (2025)
DOI: 10.1126/sciadv.adt9952

This PDF file includes:

Notes S1 to S8
Figs. S1 to S17
Tables S1 to S4
References

Supplementary Note 1: Structural and electrical properties of BMPB

The trap-state density (n_{trap}) is estimated to be 4×10^{13} cm^{-3} using the following equation:

$$n_{\mathrm{trap}} = \frac{2\varepsilon_0\varepsilon_{\mathrm{r}}V_{\mathrm{TFL}}}{qL^2} \tag{S1}$$

where ε_0, V_{TFL}, q, and L represent the vacuum permittivity, the starting bias voltage of the trap-filling region, the elemental charge, and the channel length, respectively. The relative dielectric constant (ε_{r}) of 20.7 can be obtained from the capacitance measurements (fig. S3).

In the Child's region, the *I-V* curve follows the Mott-Gurney law (*78*), and the mobility (μ) is estimated to be 2.3 cm^2 V^{-1} s^{-1} using:

$$\mu = \frac{8J_{\mathrm{D}}L^3}{9\varepsilon_0\varepsilon_{\mathrm{r}}V_{\mathrm{b}}^2} \tag{S2}$$

where J_{D} is the dark current density.

The carrier diffusion (L_{D}) is estimated to be 1.0 μm using:

$$L_{\mathrm{D}} = \left(\frac{\mu\tau k_{\mathrm{B}}T}{q}\right)^{1/2} \tag{S3}$$

where k_{B} is the Boltzmann constant and $T = 300$ K.

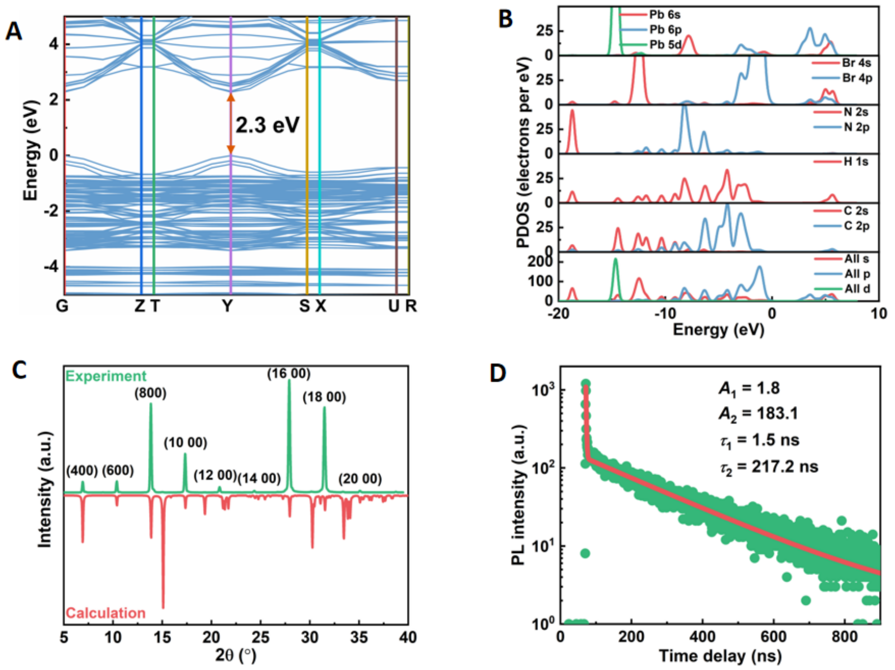

Fig. S1. Physical properties of BMPB crystals. (**A**) DFT-calculated electronic band structure. (**B**) PDOS spectra for BMPB. (**C**) XRD patterns of crystal wafer. (**D**) Time-resolved PL curve with bi-exponential fitting. The average PL lifetime (τ) is calculated to be 217.2 ns using $\tau = (A_1\tau_1^2 + A_2\tau_2^2)/(A_1\tau_1 + A_2\tau_2)$.

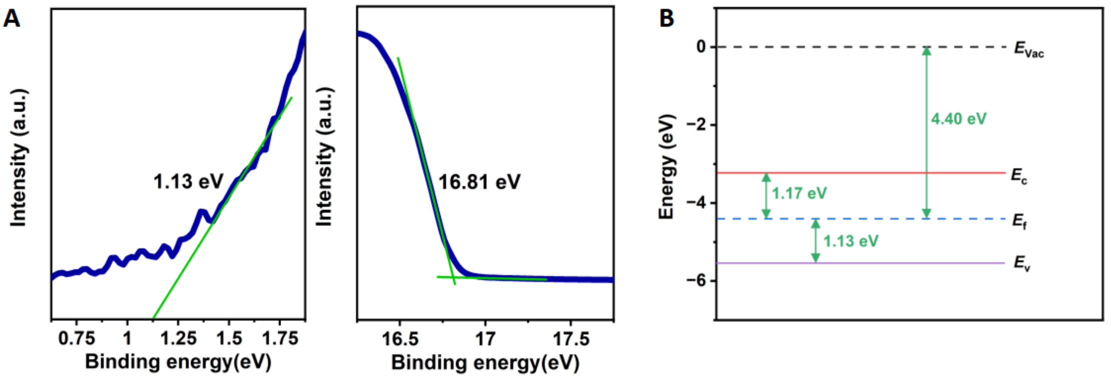

Fig. S2. UPS measurement for BMPB crystals. (**A**) UPS experimental results of BMPB. (**B**) Energy level scheme of the Fermi level and bandgap edge derived from UPS measurements and DFT calculations. E_{Vac}, E_c, E_f, and E_v represent the vacuum level, conduction band level, Fermi level, and valence band level, respectively.

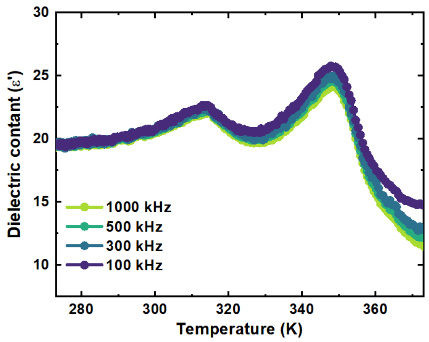

Fig. S3. Real part of relative dielectric constant (ε_r) dependence on temperature of BMPB.

Supplementary Note 2: Exciton binding energy of BMPB

The exciton binding energy can be extracted from temperature-dependent PL intensities (λ_{ex} = 405 nm, CW laser). The PL intensity dependence on temperature is shown in fig. S4 and can be fitted using the following equation (*79*)

$$I(\mathrm{T}) = \frac{I_0}{1 + Ae^{-(E_\mathrm{b}/k_\mathrm{B}T)}} \tag{S4}$$

where I_0 is the PL intensity at 0 K and E_b is the exciton binding energy.

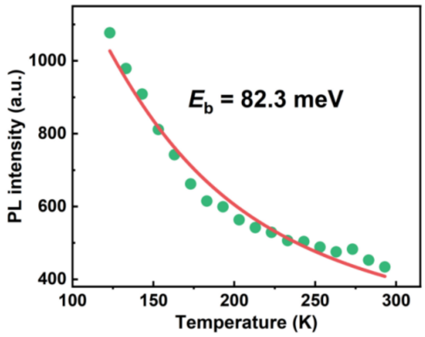

Fig. S4. Temperature-dependent PL intensities of BMPB in the temperature range of 123-293 K. The solid dots represent experimental data, and the solid lines are the fitting with Eq. S4.

Table S1. Exciton binding energy of BMPB compared with other 2D/3D perovskites.

Material	Structure	E_g (eV)	E_b (meV)	Ref.
$MAPbI_3$	Thin films	1.67-1.71 (Orthorhmobic); 1.61-1.64 (Tetragonal)	25.4±2.8 (Orthorhmobic); 12.3±2.6 (Tetragonal)	40
$MAPbBr_3$	Thin films	2.3-2.39	35±5.6	40
$MAPb_{0.8}Sn_{0.2}I_3$	Thin films	1.56	16±3	41
$CsPbI_3$	Thin films	1.72	15±1	42
$CsPbBr_3$	Thin films	2.34	33±1	42
$CsPbI_2Br$	Thin films	1.91	22±3	42

$(BA)_2(MA)Pb_2I_7$	Bulk crystal	2.5	200	43
$(C_4H_9NH_3)_2PbI_4$	Single crystal	2.77 (bulk); 2.74 (nanosheets)	180 (bulk); 190 (nanosheets)	44
p-$FPEA_2PbBr_4$	Single crystal	2.85	195	45
$(C_6H_5(CH_2)_2NH_3)_2PbI_4$	Single crystals	2.34	250	25
$(BA_{0.9}PEA_{0.1})_2(MA)_{n-1}Pb_nI_{3n+1}$	Thin film	1.66	256.5 (n=2); 243.5 (n=3); 61.7 (n=∞)	46
**BMPB**	**Single crystals**	**2.4**	**82.3**	**This work**

E_g: bandgap energy; E_b: exciton binding energy.

Supplementary Note 3: MPA-excited PL emission spectra of BMPB

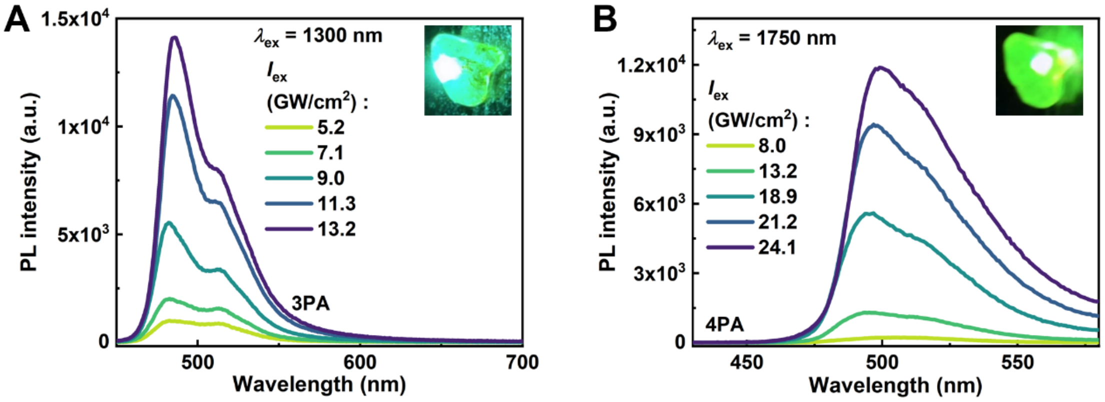

Fig. S5. MPA-excited PL spectra of BMPB at different excitation wavelengths. (**A**) λ_{ex} = 1300 nm (corresponding to 3PA); (**B**) λ_{ex} = 1750 nm (4PA).

Supplementary Note 4: Exciton-longitudinal optical-phonon coupling of BMPB

As shown in Fig. 2D in the main text, the FWHM of the 5PPL spectra continuously increases with temperature. This broadening is attributed to the coupling effect between the excitons and phonons, which can be described using the following model (*80, 81*)

$$\Gamma(T) = \Gamma_0 + \sigma T + \Gamma_{\mathrm{LO}}(\mathrm{e}^{\hbar\omega_{\mathrm{LO}}/k_{\mathrm{B}}T} - 1)^{-1} + \Gamma_{\mathrm{imp}}\mathrm{e}^{-E_{\mathrm{b}}/k_{\mathrm{B}}T} \tag{S5}$$

where Γ_0 is the inhomogeneous broadening contribution, while the second and third terms in Eq. S5 are the homogeneous broadening due to the exciton-phonon interactions, of which σ is the exciton-acoustic-phonon coupling strength, Γ_{LO} is the exciton-longitudinal optical-phonon (LO) coupling strength, and $\hbar\omega_{LO}$ is the energy of the LO phonon. Because the contribution of optical phonons to the PL broadening surpasses

that of acoustic phonons around room temperature (*82, 83*), the second term in Eq. S5 can be neglected in our fitting. The last term in Eq. S5 is due to the scattering on ionized impurities, which decreases exponentially with increasing *T*. The linear dependence of FWHM on *T* around room temperature indicates that this effect can be neglected in our case. Therefore, Eq. S5 can be simplified as follows:

$$\Gamma(T) = \Gamma_0 + \Gamma_{\mathrm{LO}}(\mathrm{e}^{\hbar\omega_{\mathrm{LO}}/k_{\mathrm{B}}T} - 1)^{-1} \tag{S6}$$

By fitting the experimental data with Eq. S6, Γ_{LO} of BMPB is derived to be 142.7 meV (Fig. 2E in the main text).

Supplementary Note 5: OA Z-scan measurement for BMPB

For a nonlinear optical effect, the MPA process can be described by the following expression

$$\frac{dI(z)}{dz} = -\alpha I(z) - \beta I^2(z) - \gamma I^3(z) - \delta I^4(z) - \varphi I^5(z) - \cdots \tag{S7}$$

where $I(z)$ is the local intensity of incident laser beam propagating along the z-axis, and z is the propagation distance of light in the medium. The parameters α, β, γ, δ, and φ are one-, two-, three-, four-, and five-photon absorption coefficients, respectively. Here, 5PA process is given as an example. At a certain photon frequency v, only the 5PA process satisfying Eq. S7 is available, then the following relation can be obtained

$$\frac{dI(z)}{dz} = -\delta I^5(z) \tag{S8}$$

and its solution is

$$I(z) = \frac{I_0}{(1 + 4\varphi z I_0^4)^{1/4}} \tag{S9}$$

when $z = L_0$, the nonlinear transmittance (T) can be expressed as

$$T = \frac{I(L_0)}{I_0} = \frac{1}{(1 + 4\varphi L_0 I_0^4)^{1/4}} \tag{S10}$$

where I_0 is the incident laser intensity and L_0 is the optical path length. Note that the

value of I_0 varies with the position z along the z-axis direction. Therefore, the transmittance T at different z can be measured experimentally by keeping the incident laser power constant. Thus, the φ value of a given medium can be determined by fitting the experimental data with Eq. S10.

Similarly, the respective absorption coefficients for 2PA-4PA can be determined based on this theory.

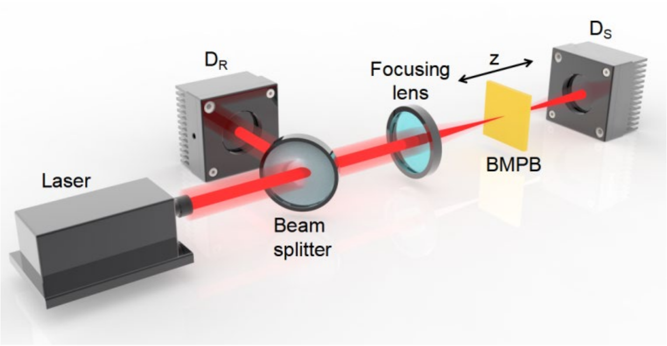

Fig. S6. Schematic diagram of the experimental setup for OA Z-scan measurements. D_S and D_R denote the signal and reference power detectors, respectively.

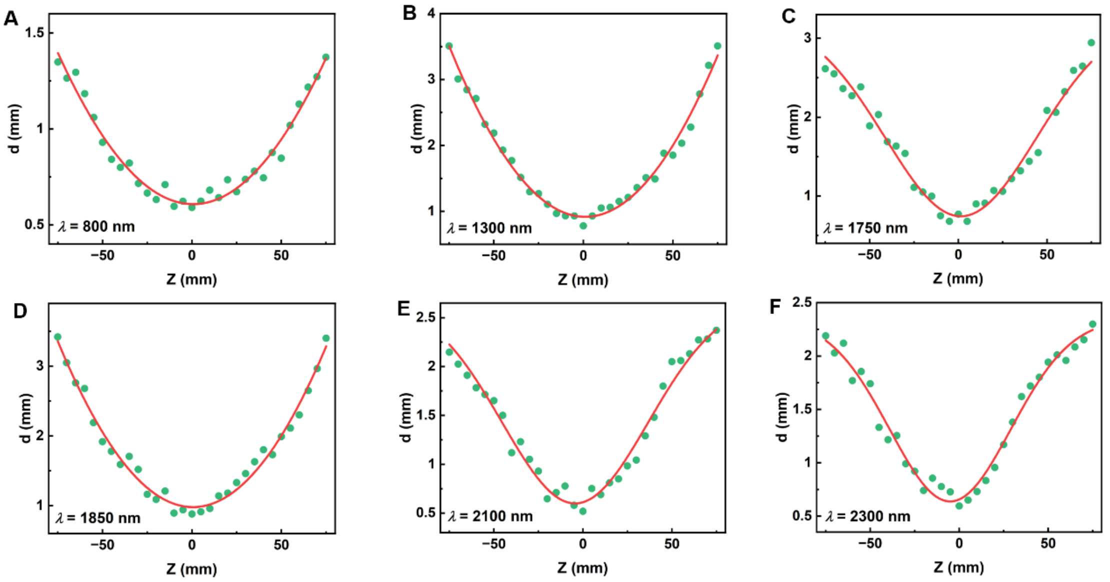

Fig. S7. Laser beam diameter (*d*) as a function of sample position (*z*) at different wavelengths. (**A**) λ = 800 nm; (**B**) λ = 1300 nm; (**C**) λ = 1750 nm; (**D**) λ = 1850 nm; (**E**) λ = 2100 nm; and (**F**) λ = 2300 nm. Solid dots: experimental data; solid lines: Gaussian fitting.

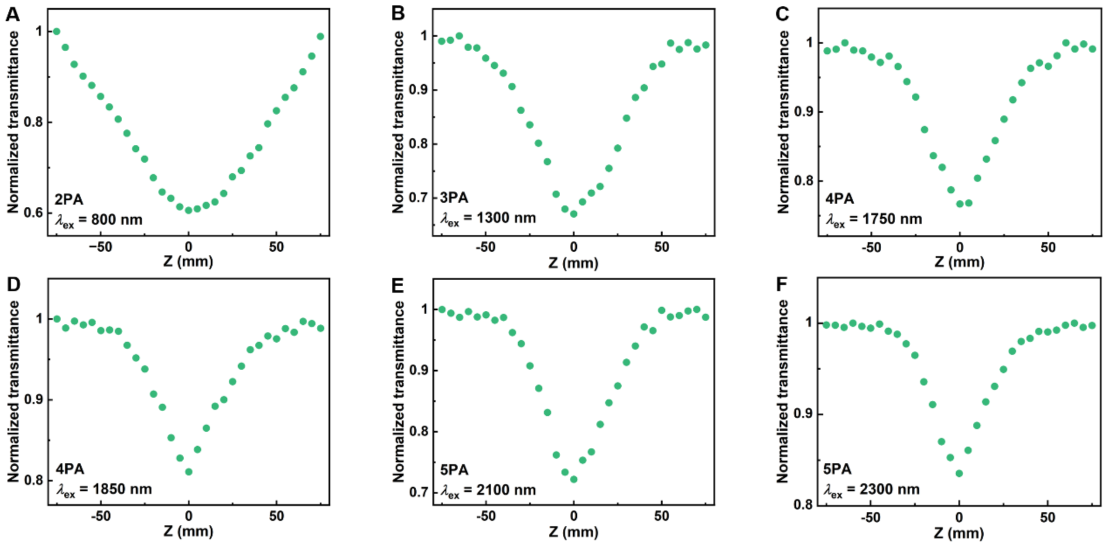

Fig. S8. Z-scan curves measured at different excitation wavelengths. (**A**) λ_{ex} = 800 nm (corresponding to 2PA); (**B**) λ_{ex} = 1300 nm (3PA); (**C**) λ_{ex} = 1750 nm (4PA); (**D**) λ_{ex} = 1850 nm (4PA); (**E**) λ_{ex} = 2100 nm (5PA); and (**F**) λ_{ex} = 2300 nm (5PA).

Table S2. Absorption coefficients for MPA processes in BMPB compared with other materials.

Material	Structure	E_g (eV)	λ_{ex} (nm)	τ (fs)	RR (Hz)	Absorption coefficient	Ref.
			**2PA process**				
$MAPbI_3$	Single crystals	1.49	1300	50	1k	β = 0.29 cm GW^{-1}	84
$MAPbCl_3$	Microcrystals	2.9	800	50	1k	β = 1.5 cm GW^{-1}	38
$FAPbBr_3$	Nanocrystals	2.29-2.38	800	35	1k	β = 0.76 cm GW^{-1}	85
$CsPbI_3$	Nanocrystals	1.79	800	100	1k	β = 0.273 cm GW^{-1}	86
$CsPbBr_3$	Nanocrystals	2.38	800	100	1k	β = 9.7×10^{-2} cm GW^{-1}	87
$CsPbCl_3$	Nanocrystals	3.03	630	100	1k	β = 7.91×10^{-3} cm GW^{-1}	88
$CsPbBr_{2.7}I_{0.3}$	Nanocrystals	2.25	800	100	1k	β = 0.338 cm GW^{-1}	86
$CsPb(Cl_{0.53}Br_{0.47})_3$	Nanocrystals	2.77	630	100	1k	β = 3.22×10^{-3} cm GW^{-1}	88
Ni-doped $CsPbBr_3$	Nanocrystals	2.19	800	70	1k	β = 9.98×10^{-2} cm GW^{-1}	89
**BMPB**	**Single crystals**	**2.4**	**800**	**150**	**5k**	**β = 2.1 cm GW^{-1}**	**This work**
			**3PA process**				
ZnS	Nanocrystals	2.8	780	120	1k	γ = 2.4×10^{-2} cm^3GW^{-2}	90
CdSe/CdS	Films	2.0	1300	100	1k	γ = 2.3×10^{-4} cm^3 GW^{-2}	91
IPPS	Organic chromophores	2.5	1197	120	1k	γ = 1.2×10^{-4} cm^3 GW^{-2}	36
$MAPbI_3$	Single crystals	1.5	1600/2100	50	1k	γ = 3.3×10^{-4}/4.8×10^{-4} cm^3 GW^{-2}	84
$CsPbCl_3$	Microcrystals	2.9	1200	50	1k	γ = 8.9×10^{-2} cm^3 GW^{-2}	37

**BMPB**	**Single crystals**	**2.4**	**1200**	**150**	**5k**	**γ = 0.11 cm^3 GW^{-2}**	**This work**
**BMPB**	**Single crystals**	**2.4**	**1300**	**150**	**5k**	**γ = 6.7×10^{-2} cm^3 GW^{-2}**	**This work**
			**4PA process**				
PRL-L3	Dye compounds	2.1	1890	160	1k	δ = 1.5×10^{-8} cm^5 GW^{-3}	34
NPH2Bz	Fluorescent probe	2.8	1600	120	1k	δ = 2.5×10^{-33} cm^5 GW^{-3}	92
$CsPbCl_3$	Microcrystals	2.9	1600	50	1k	δ = 1.1×10^{-4} cm^5 GW^{-3}	37
**BMPB**	**Single crystals**	**2.4**	**1750**	**150**	**5k**	**δ = 9.1×10^{-4} cm^5 GW^{-3}**	**This work**
**BMPB**	**Single crystals**	**2.4**	**1850**	**150**	**5k**	**δ = 6.5×10^{-4} cm^5 GW^{-3}**	**This work**
			**5PA process**				
TeO_2	Bulks	3.4	1550	100	1k	φ = 1.7×10^{-9} cm^7 GW^{-4}	35
IPPS	Organic chromophores	2.5	2100	120	1k	φ = 2.2×10^{-11} cm^7 GW^{-4}	36
$CsPbCl_3$	Microcrystals	2.9	1800	50	1k	φ = 2.3×10^{-11} cm^7 GW^{-4}	37
$MAPbCl_3$	Microcrystals	2.9	1800	/	/	φ = 3.0×10^{-8} cm^7 GW^{-4}	38
**BMPB**	**Single crystals**	**2.4**	**2100**	**150**	**5k**	**φ = 8.7×10^{-5} cm^7 GW^{-4}**	**This work**
**BMPB**	**Single crystals**	**2.4**	**2300**	**150**	**5k**	**φ = 2.2×10^{-5} cm^7 GW^{-4}**	**This work**

E_g: bandgap energy; λ_{ex}: excitation wavelength; τ: pulse duration; RR: repetition rate; β, γ, δ, and φ: 2PA-5PA absorption coefficient, respectively.

Supplementary Note 6: MPA-responsive performances of BMPB PD

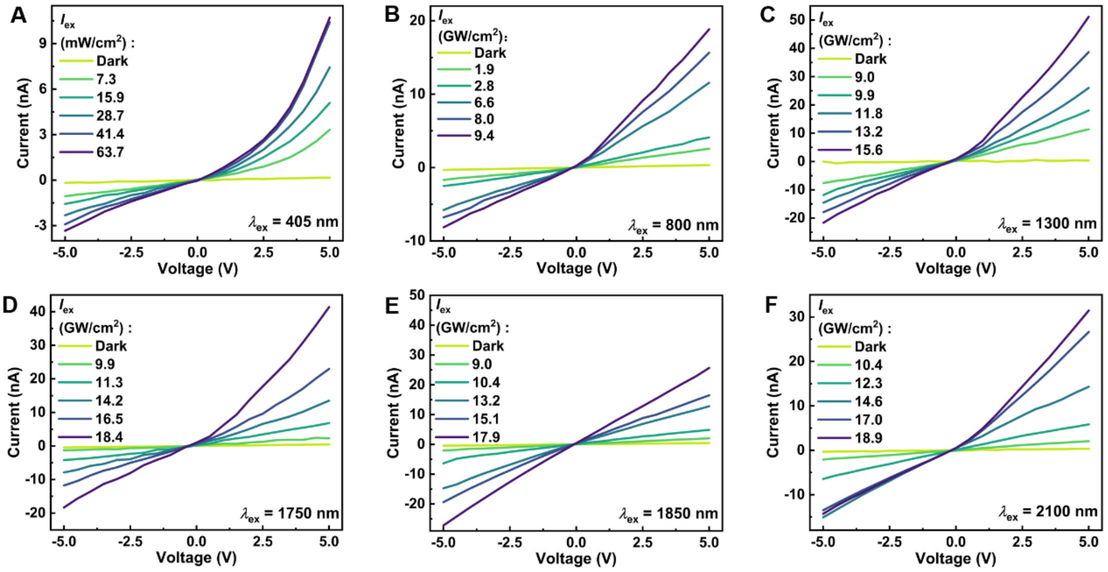

Fig. S9. *I*-*V* curves measured at different excitation wavelengths. (**A**) λ_{ex} = 405 nm (corresponding to 1PA); (**B**) λ_{ex} = 800 nm (2PA); (**C**) λ_{ex} = 1300 nm (3PA); (**D**) λ_{ex} = 1750 nm (4PA); (**E**) λ_{ex} = 1850 nm (4PA); and (**F**) λ_{ex} = 2100 nm (5PA).

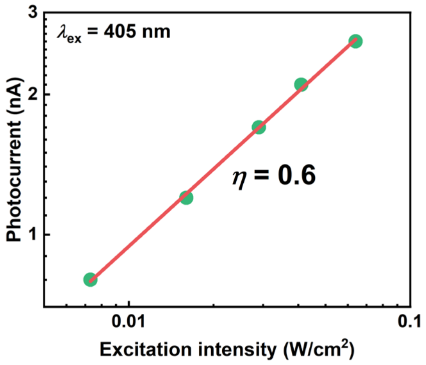

Fig. S10. Logarithmic plot of the photocurrent dependence on excitation intensity at λ_{ex} = 405 nm (V_b = 2 V). The solid dots: experimental data; solid line: linear fitting.

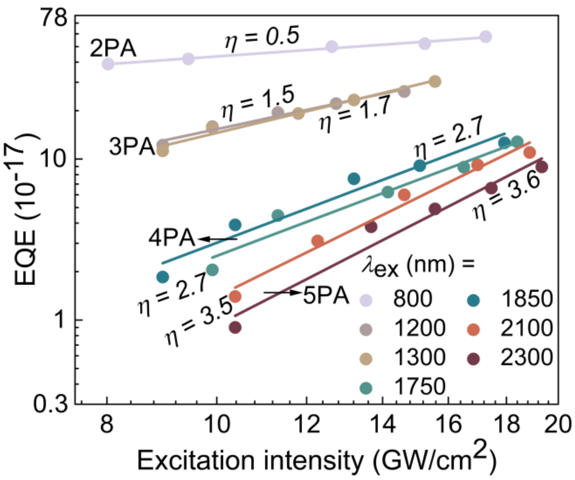

Fig. S11. Logarithmic plot of the peak-power EQE dependence on excitation intensity. Solid lines: fitting with the power-law relation $\mathrm{EQE} \propto I_{ex}^{\eta}$, where the extracted η meets $\eta \approx n-1$ for nPA.

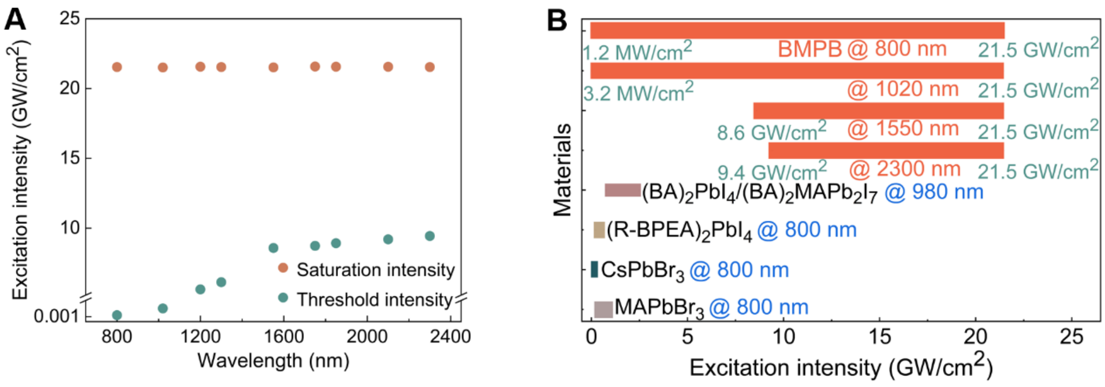

Fig. S12. Intensity-response range of BMPB PD. (**A**) Working intensity range as a function of excitation wavelength. (**B**) Intensity-response range of BMPB compared with other MPA-based PDs. References: $MAPbBr_3$ (*11*), $CsPbBr_3$ (*12*), $(R\text{-}BPEA)_2PbI_4$ (*14*), and $(BA)_2PbI_4/(BA)_2MAPb_2I_7$ (*15*).

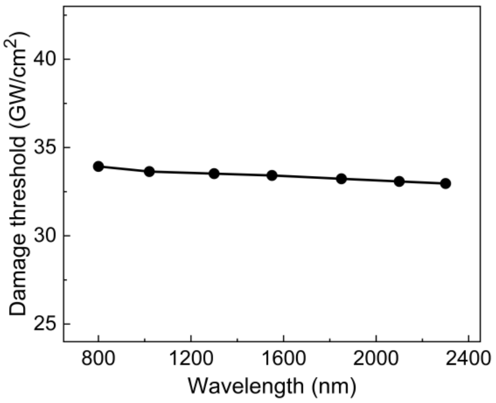

Fig. S13. Damage threshold as a function of excitation wavelength for BMPB PD.

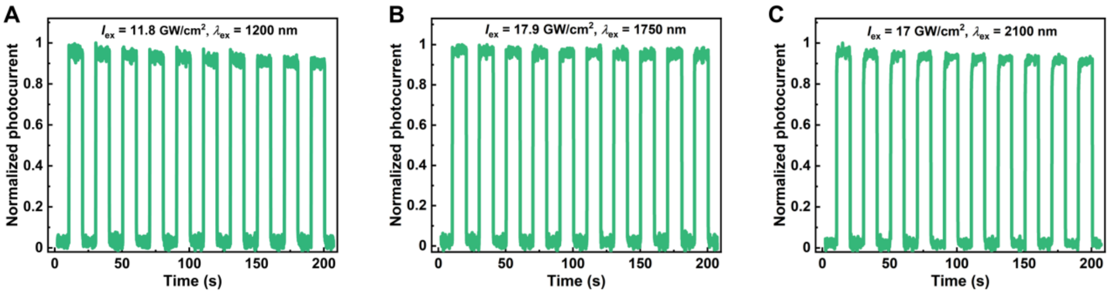

Fig. S14. On-off cycles of normalized photocurrent under periodic on-off switching of fs-laser irradiation (V_b = 2 V) at different wavelengths. (**A**) λ_{ex} = 1200 nm; (**B**) λ_{ex} = 1750 nm; and (**C**) λ_{ex} = 2100 nm.

Table S3. Intensity-response range of BMPB PD compared with other MPA PDs.

Material	Intensity-response range (GW/cm^2)	Ref.
$CH_3NH_3PbBr_3$ (@ 800 nm)	0.17-1.1	11
$CsPbBr_3$ (@ 800 nm)	3×10^{-3}-0.3	12
$(R\text{-}BPEA)_2PbI_4$ (@ 800 nm)	0.19-0.65	14
$(BA)_2PbI_4/(BA)_2MAPb_2I_7$ (@ 980 nm)	0.75-2.5	15
**BMPB (@ 800 nm)**	**1.2×10^{-3}-21.5**	**This work**
**BMPB (@ 1020 nm)**	**3.2×10^{-3}-21.5**	**This work**
**BMPB (@ 1550 nm)**	**8.6-21.5**	**This work**
**BMPB (@ 2300 nm)**	**9.4-21.5**	**This work**

Table S4. Wavelength-response range of BMPB PD compared with other MPA PDs.

Material	Wavelength-response range (nm)	Ref.
$CH_3NH_3PbBr_3$	800	11
$CH_3NH_3PbBr_3$	780	12
$CH_3NH_3Pb_{0.75}Sn_{0.25}I_3$	1064-1535	73
$CsPbBr_3$	720-900	74
$CsPbBr_3$	800-1200	12
$(C_4H_9NH_3)_2(CH_3NH_3)Pb_2I_7$	800-1600	13
$(C_4H_9NH_3)_2(CH_3NH_3)_3Pb_4I_{13}$	800-1600	13
$(R\text{-}BPEA)_2PbI_4$	800	14
$(BA)_2PbI_4/(BA)_2MAPb_2I_7$	980	15

$WS_2/(C_6H_5C_2H_4NH_3)_2PbI_4$	800	75
MoS_2	800-1050	10
ZnO	780-825	9
**BMPB**	**800-2300**	**This work**

Supplementary Note 7: MPA PD performance dependence on crystal thickness and light polarization

(1) MPA PD performance dependence on crystal thickness

According to our measurement, the thickness of the BMPB crystal above 0.1 mm has minimal influence on its MPA photodetection performance. To investigate the dependence of MPA photodetection on thickness, we fabricate five BMPB PDs with different crystal thicknesses (0.1, 0.2, 0.4, 0.6, and 0.8 mm). All devices have with identical channel area (1×5 mm^2). The photocurrents are measured under a fixed bias voltage (2 V) at two representative excitation wavelengths (800 and 2300 nm), as shown in fig. S15, A and C. Figure S15, B and D showcase the corresponding calculated peak-power responsivity (R') and the external quantum efficiency (EQE). It can be clearly seen that the MPA photodetection performance does not exhibit a significant dependence on the crystal thickness.

We infer that this phenomenon is closely related to the crystal structure of BMPB. Along the staking axis (i.e., *a*-axis), the alternative arranged organic (insulating) and inorganic (conducting) layer create a multilayered quantum well (QW) structure (the thickness of a single QW is ~ 3.3 nm), where the organic layer hinder the transport of photo-generated carriers across neighboring inorganic layers. However, for the planar-type BMPB PD, the electric field of the bias voltage and the corresponding carrier transport are oriented along the in-plane direction (i.e., *c*-axis). In this case, only a few inorganic layers near the source-drain electrodes contribute to the transport of photo-generated carriers, resulting in a minimal dependence of photo-response on the crystal thickness above 0.1 mm. However, investigating the dependence on few-layered BMPB remains challenging, as we are unable to obtain BMPB films thinner than 0.1 mm by exfoliating single crystals, due to the strong interlayer hydrogen bonds in the crystal structure, which provide high stability and resistance to fs-laser damage.

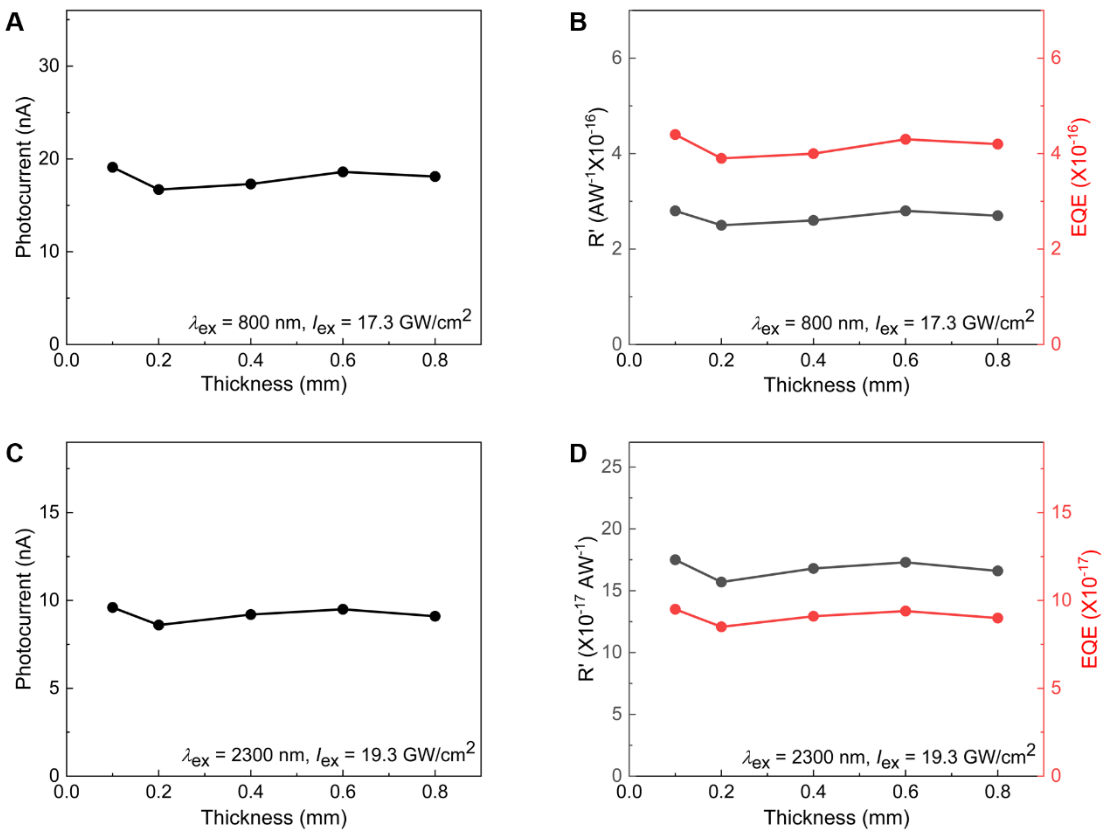

Fig. S15. MPA-excited photodetection performance dependence on the thickness of BMPB crystal. (**A** and **C**) Thickness dependence of the photocurrent in 2PA (λ_{ex} = 800 nm) and 5PA (λ_{ex} = 2300 nm) processes, respectively. (**B** and **D**) Thickness dependence of the peak-power responsivity and EQE in 2PA (λ_{ex} = 800 nm) and 5PA (λ_{ex} = 2300 nm) processes, respectively.

(2) MPA PD performance dependence on light polarization

To investigate the MPA PD performance dependence on the polarization of incident light, we measured the nonlinear optical responses in typical MPA processes, as shown in fig. S16. It is clearly illustrated that, the proposed MPA optoelectronic technique based on BMPB has no dependence on the polarization of light based on both the polarization-angle-resolved MPA data (fig. S16A) and photocurrent data (fig. S16B), respectively.

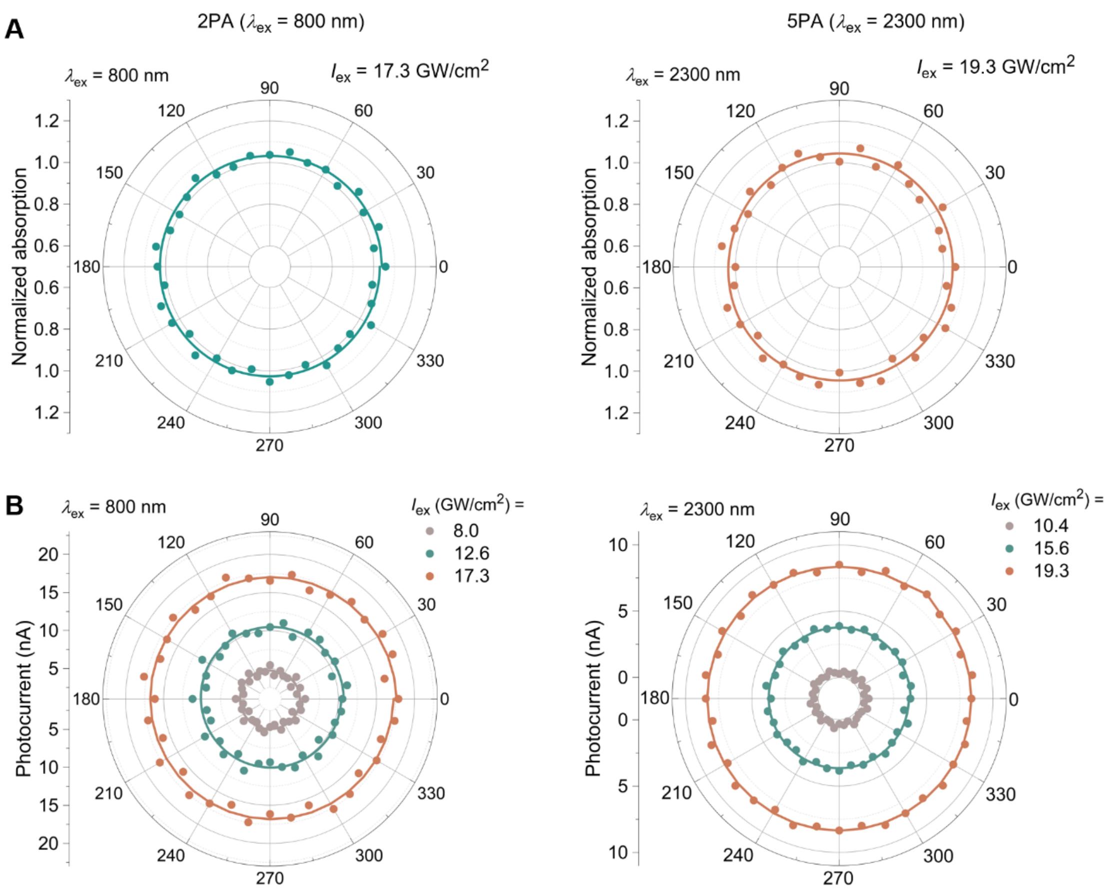

Fig. S16. Orientation evolutions of nonlinear optical responses for typical MPA processes. (**A**) Polar plots of normalized absorption in 2PA (λ_{ex} = 800 nm) and 5PA (λ_{ex} = 2300 nm) processes. (**B**) Polar plots of photocurrent at different excitation intensities in 2PA (λ_{ex} = 800 nm) and 5PA (λ_{ex} = 2300 nm) processes. 0° corresponds to the *c*-axis of BMPB crystal.

Supplementary Note 8: Spatial imaging comparison with InGaAs photodiode

Here we characterize both direct (i.e., without any attenuation) and indirect (i.e., with attenuators placed before the photodiode) spatial imaging at 900 and 1700 nm using the InGaAs photodiode (DET10D/M, Thorlabs), as shown in fig. S17. The experimental phenomena are similar to those of the commercial Si CCD or TE array camera we presented in the manuscript. For direct measurement under a fs-laser intensity of 1.4 MW/cm^2, the InGaAs photodiode exhibits strong saturation due to its low saturation threshold when directly exposed to the fs-laser beam at both excitation wavelengths. In addition, for indirect measurement as the intensity is reduced to 0.7 MW/cm^2 using additional attenuators, the high-intensity field can excite various nonlinear optical effects in the optical elements, leading to severe beam distortions.

In comparison, the BMPB PD demonstrates high imaging accuracy at 900 and 1700 nm, even under and excitation intensity of 21.5 GW/cm^2. This suggests the exceptional potential of high-performance MPA PD for accurately characterizing and imaging high-intensity broadband lasers.

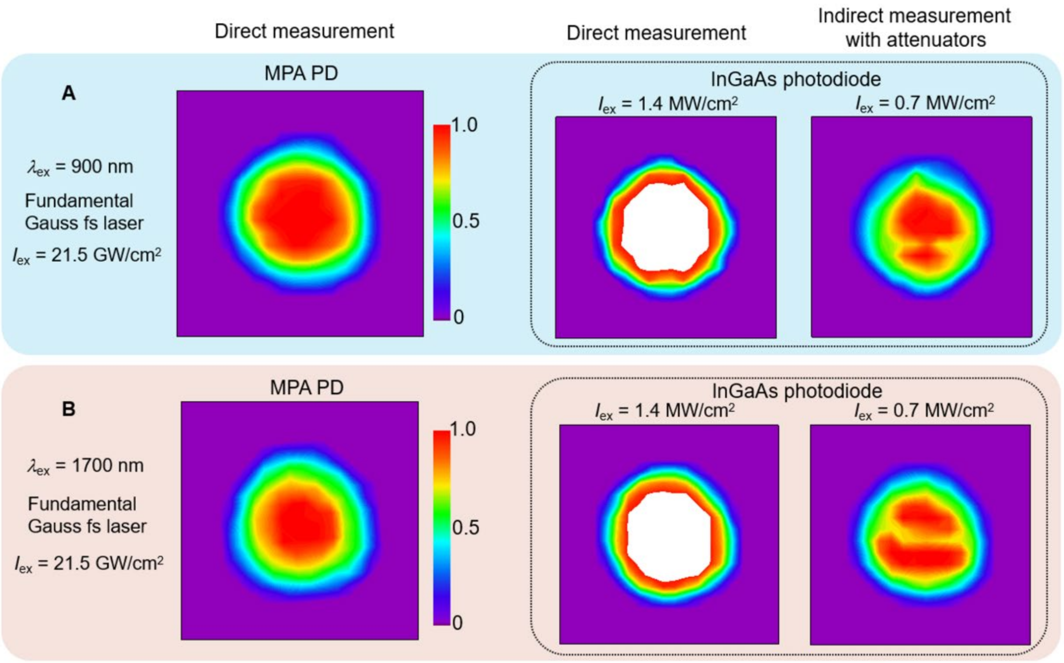

Fig. S17. Spatial imaging comparison with InGaAs photodiode. (**A** and **B**) Left: direct measurement of the fs-laser beam with fundamental Gauss mode using the MPA PD at λ_{ex} = 900 and 1700 nm, respectively. Middle: direct measurement using the commercial InGaAs photodiode at λ_{ex} = 900 and 1700 nm, respectively. Right: indirect measurement using the commercial InGaAs photodiode with additional attenuation at λ_{ex} = 900 and 1700 nm, respectively.

REFERENCES AND NOTES

1. D. E. Spence, P. N. Kean, W. Sibbett, 60-Fsec pulse generation from a self-mode-locked Ti:sapphire laser. *Opt. Lett.* **16**, 42–44 (1991).

2. K. Y. Kim, A. J. Taylor, J. H. Glownia, G. Rodriguez, Coherent control of terahertz supercontinuum generation in ultrafast supercontinuum generation in ultrafast laser-gas interactions. *Nat. Photon.* **2**, 605–609 (2008).

3. Y.-Y. Lv, J. Xu, S. Han, C. Zhang, Y. Han, J. Zhou, S.-H. Yao, X.-P. Liu, M.-H. Lu, H. Weng, Z. Xie, Y. B. Chen, J. Hu, Y.-F. Chen, S. Zhu, High-harmonic generation in Weyl semimetal β-WP_2 crystals. *Nat. Commun.* **12**, 6437 (2021).

4. C. Zhang, H. Wu, Y. Xu, J. Xu, Z. Yan, Y. Hu, Z. Xie, S. Zhu, Plasmon-assisted broadband all-optical control of highly intense femtosecond laser by weak continuous-wave laser. *Adv. Optical Mater.* **8**, 2000560 (2020).

5. W. Liu, J. Song, P. Ma, H. Xiao, P. Zhou, Effects of background spectral noise in the phase-modulated single-frequency seed laser on high-power narrow-linewidth fiber amplifiers. *Photonics Res.* **9**, 424–431 (2021).

6. J. Mauritsson, P. Johnsson, R. López-Martens, K. Varjú, W. Kornelis, J. Biegert, U. Keller, M. B. Gaarde, K. J. Schafer, A. L'Huillier, Measurement and control of the frequency chirp rate of high-order harmonic pulses. *Phys. Rev. A* **70**, 021801 (2004).

7. I. Alexeev, T. M. Antonsen, K. Y. Kim, H. M. Milchberg, Self-focusing of intense laser pulses in a clustered gas. *Phys. Rev. Lett.* **90**, 103402 (2003).

8. S. Chang, Off-axial aberrations of a Gaussian beam obliquely passing through a thinradial GRIN lens. *Optik* **126**, 4419–4423 (2015).

9. G. Lou, Y. Wu, H. Zhu, J. Li, A. Chen, Z. Chen, Y. Liang, Y. Ren, X. Gui, D. Zhong, Z. Qiu, Z. Tang, S. C. Su, Upconversion single-microbelt photodetector via two-photon absorption simultaneous. *J. Phys. D Appl. Phys.* **51**, 19LT01 (2018).

10. F. Zhou, W. Ji, Two-photon absorption and subband photodetection in monolayer MoS_2. *Opt. Lett.* **42**, 3113–3116 (2017).

11. G. Walters, B. R. Sutherland, S. Hoogland, D. Shi, R. Comin, D. P. Sellan, O. M. Bakr, E. H. Sargent, Two-photon absorption in organometallic bromide perovskites. *ACS Nano* **9**, 9340–9346 (2015).

12. B. Yang, F. Zhang, J. Chen, S. Yang, X. Xia, T. Pullerits, W. Deng, K. Han, Ultrasensitive and fast all-inorganic perovskite-based photodetector via fast carrier diffusion. *Adv. Mater.* **29**, 1703758 (2017).

13. F. Zhou, I. Abdelwahab, K. Leng, K. P. Loh, W. Ji, 2D perovskites with giant excitonic optical nonlinearities for high-performance sub-bandgap photodetection. *Adv. Mater.* **31**, e1904155 (2019).

14. Y. Peng, X. Liu, Y. P. Yao, H. Ye, X. Shang, X. Chen, J. Luo, Realization of vis-NIR dual-modal circularly polarized light detection in chiral perovskite bulk crystals. *J. Am. Chem. Soc.* **143**, 14077–14082 (2021).

15. J. Wang, Y. Mi, X. Gao, J. Li, J. Li, S. Lan, C. Fang, H. Shen, X. Wen, R. Chen, X. Liu, T. He, D. Li, Giant nonlinear optical response in 2D perovskite heterostructures. *Adv. Optical Mater.* **7**, 1900398 (2019).

16. W. Chen, S. Bhaumik, S. A. Veldhuis, G. Xing, Q. Xu, M. Grätzel, S. Mhaisalkar, N. Mathews, T. C. Sum, Giant five-photon absorption from multidimensional core-shell halide perovskite colloidal nanocrystals. *Nat. Commun.* **8**, 15198 (2017).

17. X. Zhang, S. Xiao, Z. Guo, B. Yuan, X. Wang, S. Zhang, Y. Shi, T. He, R. Chen, Improving the five-photon absorption from core-shell perovskite nanocrystals. *J. Phys. Chem. Lett.* **14**, 7581–7590 (2023).

18. Y. Jiang, K. F. Li, K. Gao, H. Lin, H. L. Tam, Y.-Y. Liu, Y. Shu, K.-L. Wong, W.-Y. Lai, K. W. Cheah, W. Huang, Frequency-upconverted stimulated emission by up to six-photon excitation

from highly extended spiro-fused ladder-type oligo(p-phenylene)s. *Angew. Chem. Int. Ed.* **60**, 10007–10015 (2021).

19. H. He, Y. Cui, B. Li, B. Wang, C. Jin, J. Yu, L. Yao, Y. Yang, B. Chen, G. Qian, Confinement of perovskite-QDs within a single MOF crystal for significantly enhanced multiphoton excited luminescence. *Adv. Mater.* **31**, 1806897 (2019).

20. J. Jiang, G. Niu, L. Sui, X. Wang, X. Zeng, Y. Zhang, L. Che, G. Wu, K. Yuan, X. Yang, Six-photon excited self-trapped excitons photoluminescence in lead-free halide perovskite. *Adv. Optical Mater.* **11**, 2202634 (2023).

21. L. Dou, Y. Yang, J. You, Z. Hong, W.-H. Chang, G. Li, Y. Yang, Solution-processed hybrid perovskite photodetectors with high detectivity. *Nat. Commun.* **5**, 5404 (2014).

22. E. A. Muljarov, S. G. Tikhodeev, N. A. Gippius, T. Ishihara, Exciton in self-organized semiconductor/insulator supperlattices: PbI-based perovskite compounds. *Phys. Rev. B* **51**, 14370–14378 (1995).

23. C. Katan, N. Mercier, J. Even, Quantum and dielectric confinement effects in lower-dimensional hybrid perovskite semiconductors. *Chem. Rev.* **119**, 3140–3192 (2019).

24. W. Chen, F. Zhang, C. Wang, M. Jia, X. Zhao, Z. Liu, Y. Ge, Y. Zhang, H. Zhang, Nonlinear photonics using low-dimensional metal-halide perovskites: Recent advances and future challenges. *Adv. Mater.* **33**, 2004446 (2021).

25. B. Cheng, T.-Y. Li, P. Maity, P.-C. Wei, D. Nordlund, K.-T. Ho, D.-H. Lien, C.-H. Lin, R.-Z. Liang, X. Miao, I. A. Ajia, J. Yin, D. Sokaras, A. Javey, I. S. Roqan, O. F. Mohammed, J.-H. He, Extremely reduced dielectric confinement in two-dimensional hybrid perovskites with large polar organics. *Commun. Phys.* **1**, 80 (2018).

26. J.-C. Blancon, A. V. Stier, H. Tsai, W. Nie, C. C. Stoumpos, B. Traoré, L. Pedesseau, M. Kepenekian, F. Katsutani, G. T. Noe, J. Kono, S. Tretiak, S. A. Crooker, C. Katan, M. G. Kanatzidis, J. J. Crochet, J. Even, A. D. Mohite, Scaling law for excitons in 2D perovskite quantum wells. *Nat. Commun.* **9**, 2254 (2018).

27. A. Shimizu, Optical nonlinearity induced by giant dipole moment of Wannier excitons. *Phy. Rev. Lett.* **61**, 613–616 (1988).

28. E. Hanamura, Very large optical nonlinearity of semiconductor microcrystallites. *Phys. Rev. B* **37**, 1273–1279 (1988).

29. E. Hanamura, Exciton enhancement of optical non-linearity in low-dimensional crystals. *Opt. Quant. Electron.* **21**, 441–450 (1989).

30. Y. Gao, N. Q. Huong, J. L. Birman, M. J. Potasek, Large nonlinear optical properties of semiconductor quantum dot arrays embedded in an organic medium. *J. Appl. Phys.* **96**, 4839–4842 (2004).

31. O. Voznyy, B. R. Sutherland, A. H. Ip, D. Zhitomirsky, E. H. Sargent, Engineering charge transport by heterostructuring solution-processed semiconductors. *Nat. Rev. Mater.* **2**, 17026 (2017).

32. H. Lu, K. Chen, R. S. Bobba, J. Shi, M. Li, Y. Wang, J. Xue, P. Xue, X. Zheng, K. E. Thorn, I. Wagner, C.-Y. Lin, Y. Song, W. Ma, Z. Tang, Q. Meng, Q. Qiao, J. M. Hodgkiss, X. Zhan, Simultaneously enhancing exciton/charge transport in organic solar cells by an organoboron additive. *Adv. Mater.* **34**, e2205926 (2022).

33. K. F. Mak, J. Shan, Photonics and optoelectronics of 2D semiconductor transition metal dichalcogenides. *Nat. Photon.* **10**, 216–226 (2016).

34. G. S. He, T.-C. Lin, S.-J. Chung, Q. Zheng, C. Lu, Y. Cui, P. N. Prasad, Two-, three-, and four-photon-pumped stimulated cavityless lasing properties of ten stilbazolium-dyes solutions. *J. Opt. Soc. Am. B* **22**, 2219 (2005).

35. K. S. Bindra, H. T. Bookey, A. K. Kar, B. S. Wherrett, X. Liu, A. Jha, Nonlinear optical properties of chalcogenide glasses: Observation of multiphoton absorption. *Appl. Phys. Lett.* **79**, 1939–1941 (2001).

36. Q. Zheng, H. Zhu, S.-C. Chen, C. Tang, E. Ma, X. Chen, Frequency-upconverted stimulated emission by simultaneous five-photon absorption. *Nat. Photon.* **7**, 234–239 (2013).

37. D. Yang, S. Chu, Y. Wang, C. K. Siu, S. Pan, S. F. Yu, Frequency upconverted amplified spontaneous emission and lasing from inorganic perovskite under simultaneous six-photon absorption. *Opt. Lett.* **43**, 2066–2069 (2018).

38. D. Yang, C. Xie, X. Xu, P. You, F. Yan, S. F. Yu, Lasing characteristics of $CH_3NH_3PbCl_3$ single-crystal microcavities under multiphoton excitation. *Adv. Optical Mater.* **6**, 1700992 (2018).

39. A. Engelmann, V. I. Yudson, P. Peineker, Enhanced optical nonlinearity of hybrid excitons in an inorganic semiconducting quantum dot covered by an organic layer. *Phys. Rev. B* **57**, 1784–1790 (1998).

40. A. M. Soufiani, F. Huang, P. Reece, R. Sheng, A. Ho-Baillie, M. A. Green, Polaronic exciton binding energy in iodide and bromide organic-inorganic lead halide perovskites. *Appl. Phys. Lett.* **107**, 231902 (2015).

41. K. Galkowski, A. Surrente, M. Baranowski, B. Zhao, Z. Yang, A. Sadhanala, S. Mackowski, S. D. Stranks, P. Plochocka, Excitonic properties of low-band-gap lead-tin halide perovskites. *ACS Energy Lett.* **4**, 615–621 (2019).

42. Z. Yang, A. Surrente, K. Galkowski, A. Miyata, O. Portugall, R. J. Sutton, A. A. Haghighirad, H. J. Snaith, D. K. Maude, P. Plochocka, R. J. Nicholas, Impact of the halide cage on the electronic properties of fully inorganic cesium lead halide perovskites. *ACS Energy Lett.* **2**, 1621–1627 (2017).

43. Y. Gao, M. Zhang, X. Zhang, G. Lu, Decreasing exciton binding energy in two-dimensional halide perovskites by lead vacancies. *J. Phys. Chem. Lett.* **10**, 3820–3827 (2019).

44. Z. Chen, Q. Zhang, M. Zhu, X. Wang, Q. Wang, A. T. S. Wee, K. P. Loh, G. Eda, Q. H. Xu, Synthesis of two-dimensional perovskite by inverse temperature crystallization and studies of exciton states by two-photon excitation spectroscopy. *Adv. Funct. Mater.* **30**, 2002661 (2020).

45. Y. Jiang, M. Cui, S. Li, C. Sun, Y. Huang, J. Wei, L. Zhang, M. Lv, C. Qin, Y. Liu, M. Yuan, Reducing the impact of Auger recombination in quasi-2D perovskite light-emitting diodes. *Nat. Commun.* **12**, 336 (2021).

46. N. Zhou, B. Huang, M. Sun, Y. Zhang, L. Li, Y. Lun, X. Wang, J. Hong, Q. Chen, H. Zhou, The spacer cations interplay for efficient and stable layered 2D perovskite solar cells. *Adv. Energy Mater.* **10**, 1901566 (2020).

47. C. V. V. Ramana, A. B. V. K. Kumar, M. A. Kumar, M. K. Moodley, Dielectric and excess dielectric constants of acetonitrile + butylamine + ethylamine, and + methylamine at 303, 313, and 323 K. *J. Chem.* **2013**, 687106 (2012).

48. M. Sendner, P. K. Nayak, D. A. Egger, S. Beck, C. Müller, B. Epding, W. Kowalsky, L. Kronik, H. J. Snaith, A. Pucci, R. Lovrinčić, Optical phonons in methylammonium lead halide perovskites and implications for charge transport. *Mater. Horiz.* **3**, 613–620 (2016).

49. Y. Li, X. Jiang, Z. Fu, Q. Huang, G.-E. Wang, W.-H. Deng, C. Wang, Z. Li, W. Yin, B. Chen, G. Xu, Coordination assembly of 2D ordered organic metal chalcogenides with widely tunable electronic band gaps. *Nat. Commun.* **11**, 261 (2020).

50. S. Prathapani, P. Bhargava, S. Mallick, Electronic band structure and carrier concentration of formamidinium-cesium mixed cation lead mixed halide hybrid perovskites. *Appl. Phys. Lett.* **112**, 092104 (2018).

51. D. Shi, V. Adinolfi, R. Comin, M. Yuan, E. Alarousu, A. Buin, Y. Chen, S. Hoogland, A. Rothenberger, K. Katsiev, Y. Losovyj, X. Zhang, P. A. Dowben, O. F. Mohammed, E. H. Sargent, O. M. Bakr, Low trap-state density and long carrier diffusion in organolead trihalide perovskite single crystals. *Science* **347**, 519–522 (2015).

52. N. Liu, P. Liu, H. Zhou, Y. Bai, Q. Chen, Understanding the defect properties of quasi-2D halide perovskites for photovoltaic applications. *J. Phys. Chem. Lett.* **11**, 3521–3528 (2020).

53. V. M. L. Corre, E. A. Duijnstee, O. E. Tambouli, J. M. Ball, H. J. Snaith, J. Lim, L. J. A. Koster, Revealing charge carrier mobility and defect densities in metal halide perovskites via space-charge-limited current measurements. *ACS Energy Lett.* **6**, 1087–1094 (2021).

54. N. Cho, F. Li, B. Turedi, L. Sinatra, S. P. Sarmah, M. R. Parida, M. I. Saidaminov, B. Murali, V. M. Burlakov, A. Goriely, O. F. Mohammed, T. Wu, O. M. Bakr, Pure crystal orientation and anisotropic charge transport in large-area hybrid perovskite films. *Nat. Commun.* **7**, 13407 (2016).

55. M. I. Saidaminov, V. Adinolfi, R. Comin, A. L. Abdelhady, W. Peng, I. Dursun, M. Yuan, S. Hoogland, E. H. Sargent, O. M. Bakr, Planar-integrated single-crystalline perovskite photodetectors. *Nat. Commun.* **6**, 8724 (2015).

56. M. I. Saidaminov, A. L. Abdelhady, B. Murali, E. Alarousu, V. M. Burlakov, W. Peng, I. Dursun, L. Wang, Y. He, G. Maculan, A. Goriely, T. Wu, O. F. Mohammed, O. M. Bakr, High-quality bulk hybrid perovskite single crystals within minutes by inverse temperature crystallization. *Nat. Commun.* **6**, 7586 (2015).

57. A. A. Zhumekenov, M. I. Saidaminov, M. A. Haque, E. Alarousu, S. P. Sarmah, B. Murali, I. Dursun, X.-H. Miao, A. L. Abdelhady, T. Wu, O. F. Mohammed, O. M. Bakr, Formamidinium lead halide perovskite crystals with unprecedented long carrier dynamics and diffusion length. *ACS Energy Lett.* **1**, 32–37 (2016).

58. Y. Wang, V. D. Ta, Y. Gao, T. C. He, R. Chen, E. Mutlugun, H. V. Demir, H. D. Sun, Stimulated emission and lasing from CdSe/CdS/ZnS core-multi-shell quantum dots by simultaneous three-photon absorption. *Adv. Mater.* **26**, 2954–2961 (2014).

59. G. S. He, K.-T. Yong, Q. Zheng, Y. Sahoo, A. Baev, A. I. Ryasnyanskiy, P. N. Prasad, Multi-photon excitation properties of CdSe quantum dots solutions and optical limiting behavior in infrared range. *Opt. Express* **15**, 12818–12833 (2007).

60. Y. Yamada, T. Yamada, L. Q. Phuong, N. Maruyama, H. Nishimura, A. Wakamiya, Y. Murata, Y. Kanemitsu, Dynamic optical properties of $CH_3NH_3PbI_3$ single crystals as revealed by

one- and two-photon excited photoluminescence measurements. *J. Am. Chem. Soc.* **137**, 10456–10459 (2015).

61. T. Yamada, Y. Yamada, H. Nishimura, Y. Nakaike, A. Wakamiya, Y. Murata, Y. Kanemitsu, Fast free-carrier diffusion in $CH_3NH_3PbBr_3$ single crystals revealed by time-resolved one- and two-photon excitation photoluminescence spectroscopy. *Adv. Electron. Mater.* **2**, 1500290 (2016).

62. T. Yamada, Y. Yamada, Y. Nakaike, A. Wakamiya, Y. Kanemitsu, Photon emission and reabsorption processes in $CH_3NH_3PbBr_3$ single crystals revealed by time-resolved two-photon-excitation photoluminescence microscopy. *Phys. Rev. Appl.* **7**, 014001 (2017).

63. K. Gauthron, J.-S. Lauret, L. Doyennette, G. Lanty, A. A. Choueiry, S. J. Zhang, A. Brehier, L. Largeau, O. Mauguin, J. Bloch, E. Deleporte, Optical spectroscopy of two-dimensional layered $(C_6H_5C_2H_4\text{-}NH_3)_2\text{-}PbI_4$ perovskite. *Opt. Express* **18**, 5912–5919 (2010).

64. H.-H. Fang, F. Wang, S. Adjokatse, N. Zhao, J. Even, M. A. Loi, Photoexcitation dynamics in solution-processed formamidinium lead iodide perovskite thin films for solar cell applications. *Light Sci. Appl.* **5**, e16056 (2016).

65. L. Yang, K. Wei, Z. Xu, F. Li, R. Chen, X. Zhang, X. Cheng, T. Jiang, Nonlinear absorption and temperature-dependent fluorescence of perovskite $FAPbBr_3$ nanocrystal. *Opt. Lett.* **43**, 122–125 (2018).

66. Y. Liang, Q. Shang, Q. Wei, L. Zhao, Z. Liu, J. Shi, Y. Zhong, J. Chen, Y. Gao, M. Li, X. Liu, G. Xing, Q. Zhang, Lasing from mechanically exfoliated 2D homologous Ruddlesden-Popper perovskite engineered by inorganic layer thickness. *Adv. Mater.* **31**, e1903030 (2019).

67. P. Cai, Y. Huang, H. J. Seo, Anti-stokes ultraviolet luminescence and exciton detrapping in the two-dimensional perovskite $(C_6H_5C_2H_4NH_3)_2PbCl_4$. *J. Phys. Chem. Lett.* **10**, 4095–4102 (2019).

68. H. Kind, H. Yan, B. Messer, M. Law, P. Yang, Nanowire ultraviolet photodetectors and optical switches. *Adv. Mater.* **14**, 158–160 (2002).

69. S.-C. Kung, W. E. van der Veer, F. Yang, K. C. Donavan, R. M. Penner, 20 μs Photocurrent response from lithographically patterned nanocrystalline cadmium selenide nanowires. *Nano Lett.* **10**, 1481–1485 (2010).

70. L. N. McLin, A case study of a bilateral femtosecond laser injury, in *ILSC 2013: Proceedings of the International Laser Safety Conference* (AIP Publishing; 2013), pp. 223–227.

71. B. A. Rockwell, R. J. Thomas, A. Vogel, Ultrashort laser pulse retinal damage mechanisms and their impact on thresholds. *Med. Laser Appl.* **25**, 84–92 (2010).

72. B. Yang, J. Chen, Q. Shi, Z. Wang, M. Gerhard, A. Dobrovolsky, I. G. Scheblykin, K. J. Karki, K. Han, T. Pullerits, High resolution mapping of two-photon excited photocurrent in perovskite microplate photodetector. *J. Phys. Chem. Lett.* **9**, 5017–5022 (2018).

73. Y. Xie, J. Fan, C. Liu, S. Chi, Z. Wang, H. Yu, H. Zhang, Y. Mai, J. Wang, Giant two-photon absorption in mixed halide perovskite $CH_3NH_3Pb_{0.75}Sn_{0.25}I_3$ thin films and application to photodetection at optical communication wavelengths. *Adv. Optical Mater.* **6**, 1700819 (2018).

74. J. Song, Q. Cui, J. Li, J. Xu, Y. Wang, L. Xu, J. Xue, Y. Dong, T. Tian, H. Sun, H. Zeng, Ultralarge all-inorganic perovskite bulk single crystal for high-performance visible-infrared dual-modal photodetectors. *Adv. Optical Mater.* **5**, 1700157 (2017).

75. Q. Wang, A. T. S. Wee, Upconversion photovoltaic effect of WS_2/2D perovskite heterostructures by two-photon absorption. *ACS Nano* **15**, 10437–10443 (2021).

76. F. Li, C. Ma, H. Wang, W. Hu, W. Yu, A. D. Sheikh, T. Wu, Ambipolar solution-processed hybrid perovskite phototransistors. *Nat. Commun.* **6**, 8238 (2015).

77. H.-C. Cheng, G. Wang, D. Li, Q. He, A. Yin, Y. Liu, H. Wu, M. Ding, Y. Huang, X. Duan, van der Waals Heterojunction devices based on organohalide perovskites and two-dimensional materials. *Nano Lett.* **16**, 367–373 (2016).

78. N. F. Mott, R. W. Gurney, *Electronic processes in ionic crystals* (Clarendon Press, 1948).

79. S. Sun, T. Salim, N. Mathews, M. Duchamp, C. Boothroyd, G. Xing, T. Z. Sum, Y. M. Lam, The origin of high efficiency in low-temperature solution-processable bilayer organometal halide hybrid solar cells. *Energ. Environ. Sci.* **7**, 399–407 (2014).

80. J. Lee, E. S. Koteles, M. O. Vassell, Luminescence linewidths of excitons in GaAs quantum wells below 150 K. *Phys. Rev. B* **33**, 5512–5516 (1986).

81. S. Rudin, T. L. Reinecke, B. Segall, Temperature-dependent exciton linewidths in semiconductors. *Phys. Rev. B* **42**, 11218–11231 (1990).

82. X. B. Zhang, T. Taliercio, S. Kolliakos, P. Lefebvre, Influence of electron-phonon interaction on the optical properties of III nitride semiconductors. *J. Phys. Condens. Matter* **13**, 7053–7074 (2001).

83. A. D. Wright, C. Verdi, R. L. Milot, G. E. Eperon, M. A. Pérez-Osorio, H. J. Snaith, F. Giustino, M. B. Johnston, L. M. Herz, Electron-phonon coupling in hybrid lead halide perovskites. *Nat. Commun.* **7**, 11755 (2016).

84. D. Yang, C. Xie, J. Sun, H. Zhu, X. Xu, P. You, S. P. Lau, F. Yan, S. F. Yu, Amplified spontaneous emission from organic-inorganic hybrid iodide perovskite single crystals under direct multiphoton excitation. *Adv. Optical Mater.* **4**, 1053–1059 (2016).

85. Z. Liu, Z. Hu, Z. Zhang, J. Du, J. Yang, X. Tang, W. Liu, Y. Leng, Two-photon pumped amplified spontaneous emission and lasing from formamidinium lead bromine nanocrystals. *ACS Photonics* **6**, 3150–3158 (2019).

86. F. Zhao, J. Li, X. Gao, X. Qiu, X. Lin, T. He, R. Chen, Comparison studies of the linear and nonlinear optical properties of $CsPbBr_xI_{3-x}$ nanocrystals: The influence of dimensionality and composition. *J. Phys. Chem. C* **123**, 9538–9543 (2019).

87. Y. Wang, X. Li, X. Zhao, L. Xiao, H. Zeng, H. Sun, Nonlinear absorption and low-threshold multiphoton pumped stimulated emission from all-inorganic perovskite nanocrystals. *Nano Lett.* **16**, 448–453 (2016).

88. J. Li, C. Ren, X. Qiu, X. Lin, R. Chen, C. Yin, T. He, Ultrafast optical nonlinearity of blue-emitting perovskite nanocrystals. *Photonics Res.* **6**, 554–559 (2018).

89. R. Ketavath, N. K. Katturi, S. G. Ghugal, H. K. Kolli, T. Swetha, V. R. Soma, B. Murali, Deciphering the ultrafast nonlinear optical properties and dynamics of pristine and Ni-doped $CsPbBr_3$ colloidal two-dimensional nanocrystal. *J. Phys. Chem. Lett.* **10**, 5577–5584 (2019).

90. J. He, W. Ji, J. Mi, Y. Zhang, J. Y. Ying, Three-photon absorption in water-soluble ZnS nanocrystals. *Appl. Phys. Lett.* **88**, 181114 (2006).

91. X. Qiu, J. Hao, J. Li, Z. Gong, S. Li, J. Cheng, X. Lin, T. He, Strong multiphoton absorption in chiral CdSe/CdS dot/rod nanocrystal-doped poly(vinyl alcohol) films. *Opt. Lett.* **44**, 2256–2259 (2019).

92. F. E. Hernández, K. D. Belfield, I. Cohanoschi, M. Balu, K. J. Schafer, Three- and four-photon absorption of a multiphoton absorbing fluorescent probe. *Appl. Optics* **43**, 5394–5398 (2004).
